# Layer-by-Layer Deposition: A Promising Environmentally Benign Flame-Retardant Treatment for Cotton, Polyester, Polyamide and Blended Textiles

**DOI:** 10.3390/ma15020432

**Published:** 2022-01-07

**Authors:** Eva Magovac, Bojana Vončina, Igor Jordanov, Jaime C. Grunlan, Sandra Bischof

**Affiliations:** 1 Department of Textile Chemistry and Ecology, University of Zagreb Faculty of Textile Technology, 10000 Zagreb, Croatia; eva.magovac@ttf.unizg.hr; 2 Laboratory for Chemistry and Environmental Protection, Faculty of Mechanical Engineering, University of Maribor, 2609 Maribor, Slovenia; bojana.voncina@um.si; 3 Department of Textiles, Faculty of Technology and Metallurgy, University Ss. Cyril and Methodius, 1000 Skopje, North Macedonia; jordanov@tmf.ukim.edu.mk; 4 Department of Mechanical Engineering, Texas A&M University, College Station, TX 77843, USA; jgrunlan@tamu.edu

**Keywords:** layer-by-layer, flame retardancy, cotton, polyamide, polyester

## Abstract

A detailed review of recent developments of layer-by-layer (LbL) deposition as a promising approach to reduce flammability of the most widely used fibers (cotton, polyester, polyamide and their blends) is presented. LbL deposition is an emerging green technology, showing numerous advantages over current commercially available finishing processes due to the use of water as a solvent for a variety of active substances. For flame-retardant (FR) purposes, different ingredients are able to build oppositely charged layers at very low concentrations in water (e.g., small organic molecules and macromolecules from renewable sources, inorganic compounds, metallic or oxide colloids, etc.). Since the layers on a textile substrate are bonded with pH and ion-sensitive electrostatic forces, the greatest technological drawback of LbL deposition for FR finishing is its non-resistance to washing cycles. Several possibilities of laundering durability improvements by different pre-treatments, as well as post-treatments to form covalent bonds between the layers, are presented in this review.

## 1. Introduction

Textiles make up one of the world’s largest industrial sectors, covering a wide range of industrial applications, such as apparel, footwear, automotive/aircraft/shipbuilding industry, civil engineering, agriculture, medicine, etc. Different industrial applications need functional properties such as flame retardancy, conductivity, magnetic shielding, antistatic and antimicrobial properties achieved by using chemicals and processes that, quite often, result in environmental pollution [1]. To reduce the consumption of water, energy and chemicals in the textile industry, the European Commission is emphasizing new industrial treatments, sustainable chemicals from renewable sources, recycling and reuse of textiles without negative impacts on fiber/fabric mechanical properties, visual appearance, wash durability or loss of any other specific property through the product life cycle [2]. In 2021, the International Association of Fire and Rescue Services reported over 19,000 deaths in the world caused by fire in buildings (91%), vehicles (8%) and other places (4%), which highlights the need for flame retardancy of fabrics [3]. In 2018, the flame-retardant (FR) market reached 2.8 million tons worldwide and the non-halogenated sector took about 31% of this total [4].

Effective FRs for textiles should fulfill the following requirements:

- Cost-effective and easy to apply in industry;
- Durable for at least 50 laundry cycles;
- Wear-resistant;
- High air/moisture permeability (comfortable and pleasant to wear),
- Should not change the appearance of fabric (color, shade);
- Should be non-toxic to humans or environment during the industrial production, usage, disposal, or fire [5].

Three types of approaches have been commonly used for obtaining flame-retardant textiles, i.e., mechanical incorporation of retardants into the filaments during extrusion, binding FR co-monomers in the process of polymerization and surface modification by FR coatings, which has become one of the most convenient, cost effective and most efficient ways to protect against fire [6]. Current commercially available FRs for finishing of the fabrics are mostly based on halogen (organo-halogen, halogen-antimony and halogen-phosphorus), phosphorus (organo-phosphorus and antimony-phosphorus), boron or metal hydroxides systems, as well as inorganic additives [7]. There are several techniques to add FR compounds onto a fabric depending on the end use, type of fabric, or material composition. Cotton and cotton blends require adding commercially available durable FRs based on tetrakis(hydroxymethyl) phosphonium salt (THPX) in a precondensate ammonia cure process and N-methylol dimethylphosphonopropionamide (N-MDMPA) derivatives in the pad–dry–cure process [8]. The drawbacks of these processes are the use of toxic ammonia in the case of the cure process and the release of formaldehyde during the production and product life-cycle in the pad–dry–cure process [9]. Where it is not possible to use THPX or MDMPA in the production process due to the environmental concerns, formaldehyde-free crosslinking agents such as butyl tetracarboxylic acid (BTCA) are used, but the resulting finish is usually only semi-durable [10]. Durable FR finishes for cotton and cotton blends are particularly important for safety clothes, while semi-durable FRs can be used for certain textile applications such as cotton fleece. FRs intended for man-made fibers, such as polyester (PES) and polyamide, can be applied to the textile by a thermosol treatment involving aqueous padding, drying and heating of the fabric. Dopents or co-reactant FRs can be added directly in melt spinning, but these additives alter the mechanical properties of polyester and polyamide fibers, thus limiting their use [11,12]. Durable intumescent FRs based on toxic halo–organic–antimony compounds can be added to all textiles by back-coating with appropriate resin [10,13]. 

Due to the very high demands on energy and water consumption, as well as the amount of chemicals used, some of them being toxic, there is a need to overcome these drawbacks by introducing new environmentally benign chemicals from renewable sources, such as chitosan, phytic acid, alginates, deoxyribonucleic acid, hydrophobins, caseins, whey proteins, etc. The traditional finishing of textile materials imparts a thick coating (~1 µm thickness) onto the fabric that results in altered properties of the coated fabrics, such as lower stiffness, significant loss of strength and poor abrasion resistance [10]. In an effort to minimize thickness of deposition and weight of textiles, several techniques, such as nanoparticle adsorption, sol–gel and dual-cure processes and layer-by-layer (LbL) deposition, are used [11,14,15,16]. 

## 2. LbL Deposition

LbL deposition dates back to the 1960s, to the invention of Iler and Kirkland, who discovered the buildup of inorganic films based on cationic boehmite fibrils and anionic silica particles [17,18]. In 1992, Decher et al. performed LbL assembly using cationic poly-[diethylmethyl(4-vinylbenzyl)ammonium iodide] and poly(allylamine hydrochloride) (PAH), as well as anionic solutions of sodium poly(styrenesulfonate) and potassium poly(vinyl sulfate) [19]. In 2013, Ariga et al. published a review on the possible application of LbL deposition in various industrial fields, including (bio)sensors, bioreactors, enzyme devices, drug delivery/release, cell coatings, solar cells, lithium batteries, photovoltaic devices, supercapacitors, transistors, color displays and gas barriers. Compounds used for LbL deposition include polyelectrolytes, inorganic nanoparticles, small derivatives based on nitrogen and phosphorus, small organic molecules, macromolecules (including biomacromolecules, such as proteins or deoxyribonucleic acid (DNA)), inorganic compounds, metallic or oxide colloids [20]. The LbL deposition process includes immersing the substrate into the solutions of oppositely charged polyelectrolytes or spraying the substrate with charged solutions [21]. In this way, it is possible to build LbL structures with the desired number of bilayers (BLs), trilayers (TLs), or quadlayers (QLs) with different functionality [22]. The interaction between opposite charges in multilayer film build-up is primarily electrostatic forces, but, today, researchers have explored donor/acceptor interactions, hydrogen bond donors/acceptors, covalent bonds, π–π interactions and stereo complex formations [23]. In conventional LbL deposition, layers are attracted by weak electrostatic forces of polyelectrolytes soluble in water, polyanions and polycations with one charged group per monomer unit, but polymers bearing hydrogen bond donors and acceptors are also able to form assemblies. These weak bonds are sensitive to environmental conditions and formed layers are easy to break. To form durable coatings, coordination polymers (inorganic or organometallic polymer structure containing metal cation centers linked by ligands) have been employed to form organic–inorganic hybrid multilayer assemblies. These complex structures can further be subjected to post-chemical reaction, such as UV or thermal curing. Unconventional methods of LbL deposition usually include two steps. The first step is forming a supramolecular complex based on various interactions (electrostatic, hydrogen-bonded, or π–π complexes, block copolymer micelles) in bulk solution. In the second step, the supramolecular complex is subsequently used as a building block for LbL assembly [24].

LbL assembly has been used to functionalize textiles with different properties, such as flame retardancy [25], conductivity [26], electromagnetic interference shielding [27], antimicrobial properties [28] and hydrophobicity [29]. These coatings can be applied on fabrics by dipping and by vertical or horizontal spraying [21]. Mateos et al. constructed a proof-of-concept automatic coating system capable of producing reproducible and precise layering with the possible industrial, large-scale application of LbL deposition by dipping [30]. Krogman et al. developed an automated system capable of depositing polymer films from atomized polyelectrolyte mists by spraying [31]. Jang et al. developed a robotic dipping system for layer-by-layer deposition of thin films [32]. Factors influencing the reproducible results are longer adsorption time and rinsing volume to avoid the cross-contamination of deposition solutions (dilution factor should be at least 1:106), as well as surface coverage of functional groups [22]. Parameters influencing the growth kinetics of LbL assembly include the type of polyelectrolytes, their molecular weights and concentrations in solutions, the pH of solutions, addition of low-molecular-weight additives or salts, additional sonication of the solutions, adsorption time and rinsing solutions [33,34,35,36]. Larger polyelectrolytes form slightly thicker coatings and require longer deposition times, while smaller molecules show strong dip time-dependent thickness of the LbL assembly [37]. Gamboa et al. investigated the influence of rinsing and drying on the growth of layers by means of an automated deposition system. In terms of thickness up to 40 BLs, the thickest films could be achieved by rinsing upward and drying upward the substrate as an intermediate step between dipping into or spraying with oppositely charged polyelectrolytes, which completely eliminated electrolyte contamination [38]. LbL deposition is a promising technique for textile finishing due to a very low concentration of active substances in polyelectrolyte solutions. Only several milligrams per milliliter are needed to obtain desired properties [39]. The purpose of this review is to present the current state of the art and future perspectives of LbL deposition applied to reduce the flammability of the most widely used textiles—cotton, polyesters, polyamides (PA6, PA 6.6) and their blends.

## 3. Thermal Degradation of Polymers

**Figure 1:**
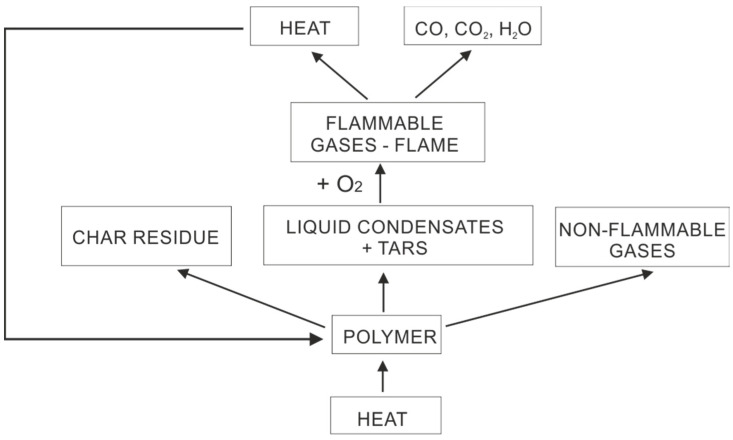
Schematic of polymer burning [5] Reproduced with permission from Magovac, E.; Bischof, S., Tekstil; published by Hrvatski inzenjerski savez tekstilaca, 2015.

In order to burn a polymeric material, thermal energy from an external source must be present to raise its temperature and initiate degradation. How much energy a given polymer absorbs depends on several factors, such as surface reflectance and absorption characteristics, and the level and the spectral characteristic of the radiant flux in the IR region. Generally, if the absorption coefficient of the polymer with respect to the external radiation is large, the temperature of the polymer surface becomes high and thermal decomposition occurs by free radical chain elimination, evolving non-combustible as well as combustible gases, which further encourage burning. These elimination reactions include random or chain end-initiated scissoring of the weakest bonds in the bridging groups connecting the aromatic rings or heterocycles, propagation and termination reactions, ending up with the formation of char in the condensed phase [40,41]. In general, the burning of polymers can be characterized by four components: heat, the oxidizing agent, the fuel (polymer) and an uninhibited chemical chain reaction. The schematic overview of polymer burning is represented in Figure 1 [5].

To prevent or to stop the fire, one or more contributors of the fire have to be removed. This is achieved by adding flame retardants with different modes of action into polymers acting chemically and/or physically in the condensed phase and/or in the gas phase [7]. Halogen-based flame retardants act chemically in the gas phase by hindering the chain-branching reactions with atmospheric oxygen, thus producing hydrogen and hydroxyl free radicals, which further propagate polymer combustion. They also dilute the flame and decrease the mass concentration of combustible gases, reducing the heat release evolved in the combustion of the gases and acting physically in the condensed phase [42]. Organophosphorus flame retardants act in the condensed phase by dehydration of polymers and char formation that acts as an insulating shield on the surface of the unburnt polymer, preventing further thermal decomposition as well as lowering the rate of transport of the combustible pyrolysis products to the flame. The process is accompanied by the endothermic release of water [43]. Flame retardants based on metal hydroxides, such as aluminum trihydroxide, release water endothermically during the decomposition to aluminum oxide acting in the condensed phase. The resulting aluminum oxide forms a shield layer, which protects unburnt polymer from burning gases in the flame [44].

### 3.1. Cotton

Among all natural fibers, cotton ranks second in textiles, with a market share of around 23% of the global fiber production in 2019 [45]. Cotton fabrics are pleasant to wear due to moisture up-take, enabled by the highly hydrophilic and reactive hydroxyl groups of glucose in cellulose [46]. Fabrics made of cotton and its blends are used for medical applications, apparel, sportwear, fashionwear and for safety clothes. However, due to its chemical composition, cotton is very flammable. The oxidative thermal decomposition of cellulose fibers starts with water desorption at ~25 °C and ends at 150 °C; it continues with cellulose dehydration between 150 and 240 °C. Above 240 °C, two parallel chemical reactions start. One is cellulose dehydration, resulting in the generation of primary char as well non-flammable gases such as water, carbon monoxide and carbon dioxide. A second chemical reaction is the depolymerization of primary char between ~240 °C and 400 °C, resulting in the generation of highly flammable levoglucosan, which, at temperatures between ~400 °C and 700 °C, yields flammable gases and initiates the generation of secondary char residue, stable at temperatures above 700 °C [47]. 

### 3.2. Polyester Fibers

Polyester fibers are the most widely used fibers worldwide, with a share of around 52% of the global fiber production in 2019 [45]. The production of polyester is low cost and the polymer is obtained by the esterification of ethylene glycol and purified terephthalic acid (TA) or dimethylterephthalate (DMT) in the presence of a catalyst. The fibers are strong and durable, dyeable, chemically and wrinkle-resistant. Fabrics made of polyester and its blends are used for apparel, sportswear, footwear, household, furniture and technical textiles, such as tire cords, car seat belts, etc. The undesirable property of polyester fibers is high flammability, with the formation of molten droplets that can easily spread the fire to other materials. Structural intermolecular changes caused by heating start near the glass transition temperature (T_g_~80 °C) and continue through melting (T_m_) between 250 and 300 °C. Above 380 °C, the thermal decomposition temperature (T_p_) of polyester occurs regardless of the type of atmosphere (vacuum, nitrogen, or air). Temperature deviations of T_g_, Tm and T_p_ are caused by deviations in molar mass, chemical composition, different catalysts used during the polyester production and the presence of additives [48]. At lower heating rates, pyrolysis takes place through four stages. In the first stage, free radical chain elimination occurs, generating methyl vinyl terephthalate and terephthalic acid. At ~280 °C, the vinyl polymerization of methyl vinyl terephthalate occurs. At ~300 °C, methyl vinyl terephthalate separates from the linear polymer chain, while forming double bonds in the linear polymer. At ~400 °C, linear polymer chain cyclization occurs [49]. 

### 3.3. Polyamide Fibers

Polyamide fibers had a market share of around 5% of the global fiber production market in 2019 [45]. The production is low cost and the polymer is obtained by the polycondensation reaction of ε-caprolactam (PA6) or the polycondensation reaction of a diamine and dicarboxylic acid (e.g., hexamethyldiamine and adipic acid—PA6.6), containing at least 85% by weight of diamine and dicarboxylic acid. Their unique properties, such as elasticity, strength, heat-, cold- and chemical resistance, makes polyamides ideal for making technical textiles such as ropes for boats, car seat belts, life vests, luggage as well as apparel such as combat uniforms, socks and swimwear [50,51]. However, PA6.6 and PA6 fibers are very flammable, with the formation of molten droplets.

The glass transition temperature of PA6 and PA6.6 fibers is between 45 and 60 °C and the melting point is between 172 and 260 °C. The range in these temperatures are also caused by deviations in molar mass, chemical composition, various catalysts used during production and the presence of additives and copolymers [52]. The decomposition temperature of polyamide also varies, but it starts, generally, between 310 and 400 °C, with a primary scission reaction of -NH–CH_2_- bonds and free radicals’ formation, followed by a complex series of secondary reactions [53]. Polyamide decomposition reactions are divided into two processes, i.e., cyclization of a part of the polymer chain of adipic acid and subsequent reactions and cleavage of the polymer chain, as well as subsequent condensation reactions (i.e., crosslinking of amine groups) [54]. The FR properties of polyamides are achieved by various organo-halogen compounds, such as trialkylphosphates and phosphonates in the form of copolymers and melamine salts [12,55,56].

### 3.4. Fiber Blends

While the behavior of cotton, polyester and polyamide is predictable during combustion, their blends react unpredictably depending on the blend ratio. During the thermal degradation of the cotton/polyester blend, cotton decomposes at ~320 °C, which is below the temperature of thermal degradation of polyester (~380 °C), thus making cotton the initial source of ignition in cotton/polyester blends. At temperatures between 250 and 260 °C, polyester melts, tending to wick on the cotton char, which results in the so-called scaffolding phenomenon [57]. Molten polyester furnishes additional fuel to the gas phase and, as the polymer temperature is raised, heat is produced from the combustion of cotton decomposition products. Additional fuel increases the vigor of gas phase oxidation [58,59]. By reducing the ratio of cotton in cotton/polyester blends, the total heat release (THR) values, as well as the char yield, increase [60,61]. Oppositely to cotton/polyester blends, by reducing the ratio of cotton in cotton/PA 6 blends, THR increases and char yield decreases [60]. This is likely due to fact that polyamide melts below the temperature of decomposition of cotton at ~256 °C, protecting the cotton until the temperature of the cotton’s decomposition reaches ~320 °C [62,63].

The gaseous products of the combustion of textiles are mostly toxic and consist, mainly, of ammonia (NH_3_), carbon dioxide (CO_2_), carbon monoxide (CO), various aliphatic and aromatic hydrocarbons, various aldehydes and acetates, hydrogen cyanide (HCN), hydrogen halides, sulfides, nitriles, nitrogen oxides (NO_x_) and nitrogen acid [64,65,66,67]. Gases found in the blood of fire victims are usually HCN and CO. Other toxic compounds generated in fires, such as hydrogen chloride (HCl), hydrogen fluoride (HF) and hydrogen bromide (HBr), belong to the category of inorganic irritants, acting as immediate corrosive agents of the surface of the respiratory tract [68]. 

## 4. Layer-by-Layer Deposition on Textiles

**Figure 2:**
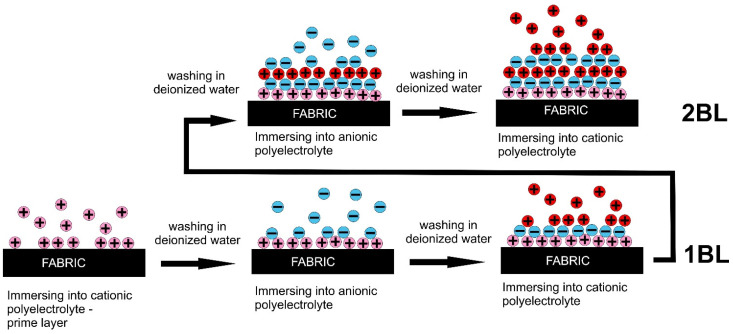
Schematic of typical layer-by-layer deposition process of immersing fabrics into oppositely charged polyelectrolytes with a primer layer.

Layer-by-layer deposition is a simple technique, able to effectively deposit active compounds on textiles. The major drawback of this technique is the poor interfacial adhesion between the textile material and polyelectrolyte solution, which depends, generally, on the hydrophilicity and surface charge of the substrate, the polyelectrolyte charge, pH and ionic strength of the solution. The general process of LbL deposition on textiles is shown in Figure 2. Before LbL deposition, the surface of textile materials should be charged enough, either positively or negatively, depending on the charge of polyelectrolytes used in the process.

Cellulose fibers are highly reactive due to the presence of surface hydroxyl groups, which enable them to react with FR compounds forming semi-durable or durable FR finishes. Polyester and polyamide fibers have a limited number of functional groups (such as -OH, -COOH, -O-CH_2_-CH_2_-, -NH_2_, etc.) on the fiber surface, which reduces the FR processability of the fabrics [69,70,71]. Even though cotton is composed of more than 90% cellulose, a negligible amount of surface-located hydrophobic waxes, pectins and proteins make raw cotton quite hydrophobic. Therefore, commercial wet processing, such as desizing to remove size, scouring to clean hydrophobic waxes, pectins and proteins and bleaching to increase the whiteness of cotton, is conducted to render cotton hydrophilic and able to accept aqueous treatment. Since wet processing of cotton is economically unfavorable due to production of effluents with high chemical oxygen demand (COD) and biochemical oxygen demand (BOD), enzymatic scouring is an excellent method to overcome these negative issues by treating the cotton at lower temperatures, for less than one hour, in near-neutral pH conditions [72,73]. 

Polyamide and polyester fibers are mainly hydrophobic due to insufficient functional groups on their surfaces [74]. Several processes have been reported to functionalize them by chemical and physical methods [75]. Chemical methods include alkaline or acid hydrolysis, having a negative environmental impact and causing damage to fabrics [76]. UV radiation and plasma activation are less harmful, but functionalization is uneven and requires complex and very expensive machinery [77,78,79,80]. Enzymatic modification of the polyamide and polyester surface is another biochemical treatment performed under environmentally benign and energy-saving conditions by using enzymes [81,82,83]. Another method for increasing the surface charge of the textiles and achieving better adhesion is applying a primer polyelectrolyte layer with an opposite charge than the substrate surface, such as branched polyethyleneimine (BPEI) or 3-aminopropyl triethoxysilane (APTES) [84]. 

### 4.1. Layer-by-Layer Deposition to Reduce Flammability of Cotton

The majority of scientific papers on the application of LbL deposition to reduce the flammability of fabrics has been devoted to cotton. The compounds used for the deposition of cotton are long-chain organic polymers [85,86,87,88,89] and short-chain organic molecules [90,91,92], as well as suspensions of inorganic nanoparticles [93,94,95]. 

One of the first studies regarding the LbL deposition of cotton with FRs was performed in 2010 by Li et al. They treated cotton fabrics with 5–20 BLs of cationic branched polyethyleneimine (BPEI) of different pH and anionic sodium montmorillonite (MMT) clay of different concentration. The study revealed that the layers became thicker by increasing the pH of the BPEI solution or the concentration of MMT clay. Cotton treated with 20 BLs of BPEI at pH 7 and 1 wt% MMT showed reduced afterglow time in vertical flame tests [85]. 

Polyethyleneimine (PEI) is a highly charged cationic polymer, rich in nitrogen, that exists in linear and branched states. The difference between these two polymers is in the type of amino group; linear PEI possesses primary and secondary amino groups, whereas branched PEI also possesses tertiary amino groups [96]. As a nitrogen rich and positively charged polymer, PEI is an excellent candidate for LbL deposition on a negatively charged, chemically bleached cotton surface. MMT is nanoclay consisting of an aluminum oxide/hydroxide layer stuck between silicate layers. Most of the clay minerals tend to have a negative charge resulting from the substitution of the silica cation (Si^4+^) by the aluminum cation (Al^3+^) in the clay sheet structure [97]. Choi et al. used 1 wt% bio-based cationic starch (CS) and anionic MMT, forming 5, 10 and 20 BLs. The LbL-coated cotton samples reduced afterglow time in vertical flame tests (VFTs) but burned completely [93]. In the majority of studies dedicated to cotton flame retardancy by means of LbL, BPEI is used either as a primer layer for better adhesion of chemical compounds to the cotton surface or as one of the oppositely charged pairs for a bilayer recipe. 

**Figure 3:**
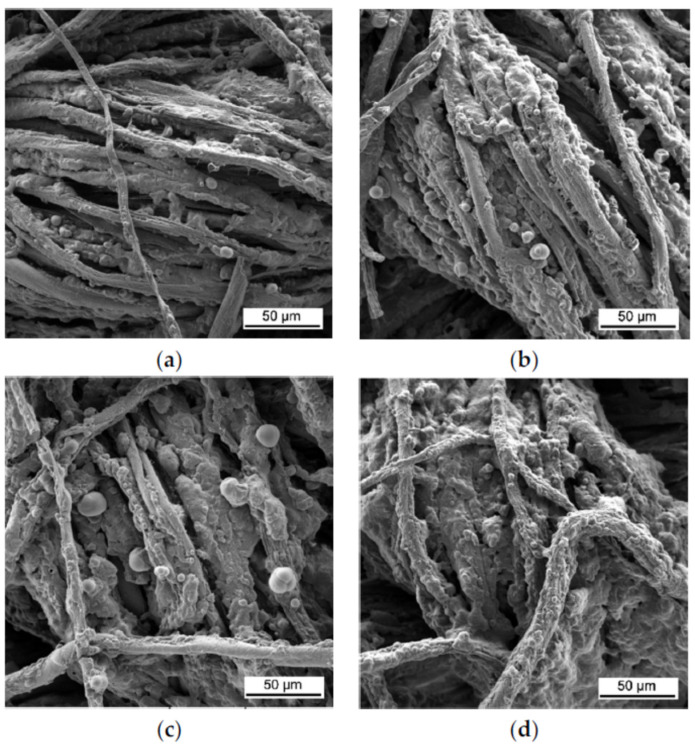
SEM images of the char of cotton samples treated with (**a**) 8, (**b**) 10, (**c**) 12 and (**d**) 15 BLs of PA/CH-U after performing vertical flame testing [92].

Another chemical compound used either as a primer layer or as one of the oppositely charged polyelectrolyte is a positively charged coupling agent rich in nitrogen and silicon 3-aminopropyl triethoxysilane (APTES), which is mostly used as a sol–gel precursor in the preparation of sol–gel materials and coatings [98]. Li et al. coated cotton fabric with 5, 10 and 15 BLs of 5 wt% cationic APTES and 2 wt% anionic phytic acid (PA), with drying at 100 °C after each dipping step. The 15 BL treatment was able to stop fire immediately after removing the ignition source [99]. PA is an inexpensive and easily obtained phosphorus-rich chelating agent from plant/seed sources, with high absorption of polycationic compounds [100]. In 2019, the same group of authors used a 1 wt% cationic suspension of PEI with alumina-coated silica nanoparticles (SiO_2_) at pH 5 (instead of APTES) and anionic 2 wt% PA at pH 6 forming two, four and seven BL. The minimum number of BLs passing VFTs was 2, with the limiting oxygen index (LOI) value of 26.0%. The study also showed a minor loss in breaking strength in the warp and weft directions (~15%) relative to untreated cotton, likely caused by the formation of hydrogen bonds between SiO_2_–PEI/PA and cotton fibers and the breaking of intermolecular and intramolecular hydrogen bonds in cellulose. The minor reduction in the loss of breaking strength after LbL treatment is an advantage over the commercial processes, which exhibit higher loss in mechanical strength [94]. The same authors used 4 wt% anionic polyphosphoric acid (PPA) instead of PA, thus reducing the number of BLs from two to one with the same FR and mechanical performance [101]. Anionic PA, as a green alternative to commercial phosphorus-based FR for cotton, has been used by several authors. Liu et al. coated cotton with a cationic solution of 0.5 wt% PEI, with added low-molecular-weight 2.0 wt% melamine (ME) at pH 4 in combination with 3.0 wt% PA at pH 4, forming two and four BLs. Only the samples coated with four BLs passed VFTs [90]. In another study 5, 10 and 15 QLs of cationic 5.0 wt% APTES, 2 wt% anionic PA, 1 wt% cationic chitosan (CH) and again anionic PA (the pH of all solutions was 3.5) were deposited with subsequent dipping and drying at 100 °C. The LOI value fabric coated with 15 BLs was 29.0% and it passed VFTs [86]. CH is another positively charged polymer from renewable sources (shells of shrimp and other sea crustaceans) widely used for LbL deposition due to its pKa value of 6.5 from amino group in a linear polysaccharide molecule composed of β-(1-4)-linked d-glucosamine (deacetylated unit) and N-acetyl-d-glucosamine (acetylated unit) [102]. Chen et al. pre-treated cotton by immersing it into positively charged APTES to improve the adhesion of two, three and six BLs consisting of polyanionic ammonium polyphosphate (APP, 1 wt%) and polycationic CH (0.5 wt%). Each immersion step was followed by drying at 100 °C. Only three and six BLs passed VFTs [87]. Anionic PA (2 wt%), with added sulfonated melamine-formaldehyde (SMF, 1 wt%), was used in the study of Pan and Zhao in 2018. Cotton fabric was coated with 5 and 10 BLs of cationic CH (0.5 wt%, pH 5) and anionic PA–SMF (pH 5). Only fabric treated with 10 BLs passed VFTs [91]. Zhang et al. successfully coated cotton with eight BLs of positively charged PEI (0.5 wt%, pH 9) and negatively charged PA (2.0 wt%, pH 4) by dipping in deionized water (DI) and drying after each dip. The coated fabrics passed VFTs [88]. Zilke et al. pre-treated cotton fabric with BPEI (1 wt%) to add a positive charge on the fabric surface and then dipped it into anionic PA (5 wt%, pH 0.7) and cationic 5 wt% polyvinyl amine (PVAm), forming 5, 10 and 15 BLs. The lowest number of BLs passing horizontal flame spreading test was 10 [89]. PVAm is a linear polymer with the highest content of primary amine functional groups of any polymers [103]. Magovac et al. used 8, 10 and 12 BLs of anionic PA (2 wt%, pH 4) and cationic CH (0.5 wt%) with added urea (U, 10 wt%), where 10 BLs passed VFTs and the char left after performing the test showed characteristic intumescent bubbling, as shown in Figure 3 [92]. 

**Figure 4:**
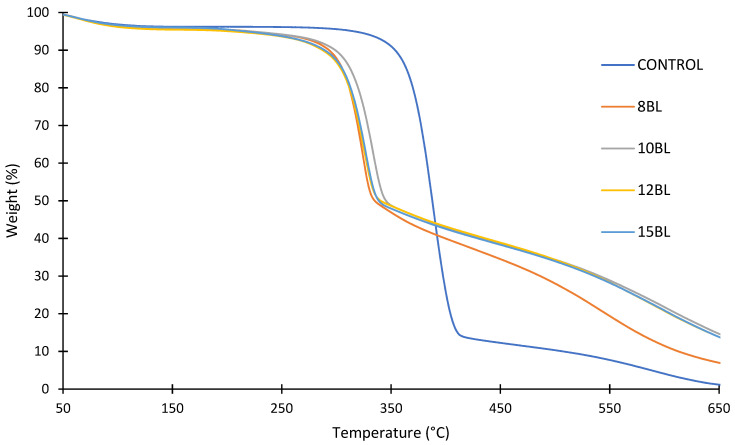
TGA of untreated and treated cotton fabrics with 8, 10, 12 and 15 BLs of PA/CH-U [92].

The thermogravimetric analysis (TGA) showed an increase in char with the increase in bilayers at 650 °C (from 1.2% for untreated cotton to 7.0% for 8 BLs and 14.1% for 10, 12 and 15 BLs), as well as the reduction in degradation temperatures, as shown in Figure 4 [92]. CH-U/PA decreased the decomposition rate of cotton by generating more non-flammable gases (e.g., CO, CO_2_, NO_x_) instead of highly flammable levoglucosane, which diluted the concentration of the combustible gases and absorbs heat, causing bubbling. At the same time, urea catalyzed the reaction of PA, as well as the decomposition of cellulose at lower temperature, thus forming intumescent char, which acted as a physical barrier that blocked heat and oxygen [104].

In 2020, Liu et al. studied the reduction in the flammability of cotton by using fully environmentally benign compounds—egg white protein and PA. Egg white protein is rich in amino acids, phosphorus, sulfur and metal complexes, such as calcium, iron, etc. Due to its complex nature, the protein attracts negatively charged PA. The study showed that even one BL of egg white protein and PA (70 wt%) gave excellent FR performance. TGA in air demonstrated a char yield of almost 33% at 600 °C and microscale combustion calorimeter (MCC) data revealed a 23% reduction in pHRR and a 67% reduction in THR [105].

Another high charge density cationic nitrogen-rich polymer used in LbL polyelectrolyte pairs is polydiallyldimethylammonium chloride (PDAC), widely used for coagulating and removing negatively charged particles and dissolved organic matter from drinking water [106]. Carosio et al. used a 1 wt% cationic solution of PDAC, 1 wt% anionic poly (acrylic acid) (PAA) and anionic 1 wt% suspension of APP. Cotton treated with a combination of PDAC/PAA/PDAC/APP in 1, 5 and 10 quadlayers (QLs), passed the horizontal flame test (HFT) [107]. In their second study, instead of APP, they used nitrogen and phosphorus-rich anionic 1 wt% DNA, forming 5 and 10 BLs. Such treated cotton was then immersed into 0.1 wt% and 1 wt% hydrotalcite (HT) nanoparticle suspensions. HT is a zeolite that is used as an antacid in medicine. The 0.1 wt% HT concentration lowered the minimum number of BLs required for obtaining cotton self-extinguishment. All samples passed the HFT, but the 0.1 wt% HT concentration showed the best performance in terms of pHRR (33%) and THR reductions (27%), relative to untreated cotton [108]. Jang et al. used 0.25 wt% cationic polyvinyl alcohol (PVA) and a 0.1 wt% anionic suspension of graphene nanoplatelets (GNP) and poly (4-styrenesulfonic acid) (PSS) to build 10 BLs on cotton. It resulted in a reduction in pHRR by 34.4% and in THR by 47.4%, respectively, but did not pass VFTs [95]. 

Another interesting study made by Pan et al. was to investigate the effect of barium, nickel and cobalt ions crosslinked with alginate on the thermal stability and flammability of cotton fabric. Cotton was coated with PEI (0.5 wt%, pH 9) and anionic sodium alginate (SA; 0.3 wt%, pH 7), forming 10 BLs. The samples were then immersed into 5 mol/L solutions of metal salts, such as barium chloride (BaCl_2_), cobalt acetate tetrahydrate (C_4_H_6_O_4_Co x 4H_2_O), or nickel acetate tetrahydrate (C_4_H_6_O_4_Ni x 4H_2_O), for crosslinking. Adding metal ions led to improved char residue and reduced DTG peak compared to untreated cotton, as well as the reduction in HFT burning rate. In terms of durability of treatment, the metal crosslinked LbL coating was durable even up to 6 h of washing in a detergent solution [109]. Alginate is a linear polysaccharide consisting of a-l-guluronic acid and β-d-mannuronic acid residues produced by brown algae and bacteria. It is used in the food industry as a thickening agent, gelling agent, emulsifier, stabilizer and texture-improver, as well as in medicine for wound dressing [110]. Pan et al. continued their studies on reducing the flammability of cotton and, instead of anionic alginate, they used a 2 wt% anionic hypophosphorous acid-modified chitosan (HACH) solution, forming 5 and 10 BLs of PEI/HACH. The cotton fabric was then immersed into a 1 wt% solution of genipin, a natural cross-linker for chitosan, proteins, collagen, etc. Ten bilayer coatings extinguished the fire of cotton during the horizontal burning test. PHRR and THR of 10 BL fabrics were reduced by 73% and 80%, comparing with those of pure cotton. Cotton coated with 10 BLs and cross-linked with genipin exhibited FR properties up to two washing cycles [111]. 

Wang et al. coated cotton fabrics with a 1 wt% cationic solution of CH with added p-aminobenzene sulfonic acid-modified melamine (AMM, 3.3 wt%) and 3.3 wt% anionic APP to build 5, 10 and 15 BLs. The resulting 15 BLs on cotton fabric exhibited excellent FR properties (LOI 31.5%, 40% decrease in pHRR, 60% THR reduction with 24.1 wt% char residue and passed VFTs), as well as showing low cytotoxicity in a cell culture [112]. In 2020, Lazar et al. pre-treated cotton fabric with a 1 wt% solution of PEI to generate a positive charge on a cotton surface and then coated it with 5, 10 and 15 BLs of 2 wt% anionic PSP (pH 4) and 0.1 wt% cationic CH (pH 4). Each dipping step was followed by rinsing in deionized water. The resulting FR cotton fabric passed VFTs. By adding a 100 mM solution of tris(hydroxymethyl)aminomethane (THAM, pH 4) to the rinsing steps, the number of bilayers needed to achieve the same FR properties of treated cotton fabrics passing VFTs was reduced to 10 [113]. Table 1 gives a review of polyelectrolytes used to achieve FR of cotton fabrics.

Most of the studies dedicated to cotton flame retardancy by means of LbL deposition show that compounds consisting of nitrogen, phosphorus, sulfur or inorganic particles reduced the pHRR values from 23% to 73% and the THR values from 27% to 80%, relative to untreated cotton. There is no general rule regarding how to obtain an optimal combination of FR ingredients to achieve a self-extinguishing behavior of cotton comparable to commercial flame-retardant finishing. The FR properties of LbL-treated cotton depended on the ionic strength of charged layers, number of layers, chemicals used, concentration of polyelectrolytes in solution, pH of solution, dipping time, pre-treatment of cotton fabrics and post-treatment of treated fabrics. By increasing the number of layers, as well as the concentration of chemical compounds in charged solutions (up to 10 wt%), the thermal stability of cotton increased. In terms of durability of LbL treatments, only a few studies show that the durability of FR on cotton was poor and the chemicals were washed away. The durability of FR treatment up to two laundry cycles in water or detergent solution was improved by post-curing with a crosslinking agent [111]. 

### 4.2. Multifunctional Finishing of Cotton

Besides excellent flame retardancy, cotton fabrics should very often satisfy many other properties, such as antistatic, antibacterial properties, hydrophobicity, self-cleaning and antifouling properties, wrinkle resistance, UV protection, electromagnetic interference shielding and conductivity [114]. The multifunctionality of cotton is possible to achieve via LbL deposition of commercially available compounds or simply by immersion of LbL-treated cotton into an active substance as a post-treatment. The wash durability of such multifunctional properties could be much better with crosslinking performed during thermal curing. An et al. coated cotton first with PEI (0.01 M) as a primer and then with 5 mg/mL of a graphene oxide (GO) anionic suspension and 3 wt% cationic caprolactam modified casein emulsion (CA), forming 1, 5 and 10 BLs. The final step was immersion into a sodium borohydride (NaBH_4_) solution, followed by APP (7 wt%) solution. The obtained LOI of this treated cotton fabric (10 BLs) was 23.6%, passing VFTs, whereas pHRR was reduced by 64% and THR was reduced by 38%, relative to untreated cotton. These fabrics also obtained excellent antistatic properties [115]. Zang et al. coated cotton fabrics with 10 QLs (two-sided) of cationic APTES (5 wt%, pH 4), anionic SA (3 mg/mL, pH 7), anionic APP (1 wt%, pH 9) and different concentrations of anionic GO (0.5; 1; 1.5 mg/mL) by spraying, which resulted in a fabric with improved FR, antistatic and antibacterial properties. Among all the treated fabrics, cotton treated with a 1 mg/mL concentration of anionic GO had the best self-extinguishing performance, with a pHRR value of about 3.2% of uncoated cotton (the reduction in pHRR was ~96.8% and that in THR was ~93%) [116]. 

Another interesting study dealing with FR and the antimicrobial properties of LbL-coated cotton comes from Li et al. Cotton fabrics were first pre-treated with 1 wt% cationic PEI as a primer and then coated with 10, 20 and 30 BLs of an anionic PA solution (2 wt%, pH 4) and a 1 wt% cationic poly[3-(5,5-cyanuricacidpropyl)-siloxane-co-trimethylammoniumpropylsiloxane chloride (PCQS) solution. At 30 BLs, the break strength of the treated cotton decreased by 14% in the warp and by 6% in the weft direction. The fabric that passed VFTs (30 BLs), with an LOI value of 29.8%, was then immersed into a 0.5 wt% antibacterial sodium hypochlorite solution (NaClO) at pH 7. The strength of cotton slightly decreased after chlorination [117]. In 2020, the same researchers treated cotton with one BL of a cationic CH solution and a 3 wt% anionic ammonium phytate (AP) solution (pH 7) to reduce the number of BLs, thus obtaining fabric with efficient FR as well as antimicrobial properties. The LOI value of this treated cotton was 27%, with a char length of 78 mm after performing VFTs [118]. In 2021, Magovac et al. used a 2 wt% anionic PA solution (pH 4) and 0.5 wt% cationic CH solution (pH 4) with added U (10 wt%) to form 8, 10 and 12 BLs. At the end of the layering, the FR-treated cotton fabric was immersed into a 2% CuSO_4_ solution to achieve additional antibacterial property. The results showed that cotton treated with 12 BLs self-extinguished the flame in VFTs (char length, 6.5 cm), with an LOI value of 26% and a reduction in pHRR by 62% and in THR by 54%. The resulting cotton killed almost 100% of bacteria [104].

Li et al. coated cotton fabric with cationic PEI with added SiO_2_ and anionic PPA (1 BL), as mentioned in Section 4.1. This fabric was finally treated with a commercially available water-repellent finish (6 wt%, pH 6) by a dip–pad–dry process. The resulting fabrics passed VFTs and exhibited excellent hydrophobic properties [101]. Excellent FR, as well as hydrophobic properties, could be achieved by coating cotton with four BLs of cationic PEI with added melamine (ME) and anionic PA, then immersing into chloroform solution and curing [90].

Another interesting study was conducted by Lin et al. by coating cotton fabric with only one BL of cationic BPEI (2 mg/mL) and anionic APP (80 mg/mL) and immersing it into polydimethyl siloxane (PDMS) with fluorinated silica (F-SiO_2_), followed by crosslinking in an oven at 130 °C for 30 min. This fabric exhibited excellent FR (the sample passed VFTs; pHRR and THR values were reduced by 86% and 39%), hydrophobic, self-cleaning, self-healing and antifouling properties with acid/alkali resistance [119]. The same properties could be achieved by coating cotton fabric with 16 BLs of cationic poly (dimethyldiallyl) ammonium chloride (PDDA) and anionic boron nitride nanosheets (BNNS) [120]. To obtain FR, wrinkle-resistant, antibacterial and UV-protective properties of cotton fabric, Safi et al. dipped fabrics first into a cationic solution consisting of 1 wt% CH, 5 wt% citric acid and 2 wt% sodium hypophosphite (SHP), followed by drying at 110 °C; then, they dipped them into a solution of 5 wt% sodium lignin sulphonate (SLS) and 4 wt% boric acid (BA), followed by drying at 80 °C for each dip. At the end, the fabrics were cured at 150 °C for 4 min. Three bilayers passed VFTs with an LOI of 30.5, while the pHRR was reduced by 50% relative to untreated cotton [121].

In a recent study by Xue et al., cotton fabric was coated with 10 BLs of cationic BPEI and anionic carbon nanotubes (CNT). Ten bilayers were then immersed into BPEI and APP, forming one BL. Each dipping was followed by drying at 60 °C. The resulting multilayered fabrics exhibited excellent FR (char length after combustion in VFTs was 7 cm) as well as conductive properties. By immersing the same fabric into one trilayer (TL) consisting of BPEI/APP/PDMS instead of BPEI/APP and subsequent drying and curing at 60 °C, the resulting fabric became superhydrophobic, acid/alkali/organic solvent-resistant, UV-protective and wash-resistant after long-time laundering [122]. To obtain excellent FR properties, conductivity and electromagnetic interference shielding durable under continuous external forces or washing tests, Zhang et al. treated fabrics with eight BLs of PEI and PA followed by drying after each dip (already mentioned in Section 4.1). The coated fabric was then immersed into a 0.8 wt% ethanol suspension of silver nanowires (AgNWs) from one to four times, followed by drying at 50 °C after each immersion step. Only four-time-immersed treated cotton suppressed flames, passing VFTs with an LOI value of 37% and a reduction in pHRR and THR by 41.4% and 27.1%, respectively [88]. Table 2 reviews polyelectrolytes used to combine FR with other functional properties and achieve the multifunctionality of cotton fabrics.

Studies dedicated to LbL deposition for multifunctional finishing of cotton show an unlimited choice of chemicals at a very low concentration (up to 10 wt%) applied either as layers or by immersing treated cotton into a charged solution at the end of the deposition. This multifunctional finishing reduced the pHRR values by 41–97% and the THR values by 27–93%. By combining different chemicals and varying their concentrations as well as number of layers, it was possible to achieve multifunctional cotton with LOI values greater than 29% [117,121]. A few studies show that LbL deposition had minimal influence on the break strength of treated cotton (at a certain number of layers, the break strength decreased by 14% in the warp and by 6% in the weft direction) [117]. The major problem of LbL multifunctional finishing is wash durability, which could be improved by post-curing with an appropriate crosslinking agent [88,119,122].

### 4.3. Layer-by-Layer Deposition to Reduce Flammability of Polyester

There are relatively few studies regarding the use of layer-by-layer deposition to reduce the flammability of polyester [107,123,124,125,126,127,128,129,130,131]. The compounds used for the treatment of polyester are long-chain organic water-soluble polymers (polyelectrolytes), short-chain organic molecules and suspensions of inorganic nanoparticles. For a better adhesion of these compounds to polyester, or as one of the oppositely charged pair of a BL, long-chain organic water-soluble charged polymers, such as PDAC, PAH, CH and PEI/BPEI, have been used [107,123,125,126,127,128]. Another option to increase the charge of polyester fabric is surface functionalization, such as alkali hydrolysis and UV-grafting [130].

Carosio et al. used a 1 wt% positively charged PDAC solution, widely utilized for building LbL assemblies in combination with 1 wt% negatively charged PAA, capable of crosslinking at temperatures above 200 °C, as well as a 1 wt% negatively charged APP suspension. Polyester fabrics treated with a combination of PDAC/PAA/PDAC/APP in 1, 5 and 10 quadlayers (QLs), limited the flammability of the fabrics by suppressing the afterglow and melt dripping, as well as lowering heat release during combustion [107]. In another study, three different 0.2 wt% cationic suspensions compounds—PDAC, SiO_2_ and polyhedral oligomeric silsesquioxane cage molecules carrying eight n-propylammonium chloride groups (POSS^®^)—with a 0.2% anionic suspension of α-zirconium phosphate (ZrP) were deposited as 5 and 10 BLs. The treated fabric showed an overall improvement in thermal stability by increasing the time to ignition (up to 86% for PDAC) and decreasing the pHRR (up to 26% for POSS). Alumina-coated silica nanoparticles reduced the production of smoke (up to 25%), but no VFT was performed [123]. The same group of authors studied the influence of LbL spraying vs. dipping on the flammability of fabric. Polyester was coated with five BLs of a cationic 0.2 wt% suspension of alumina-coated silica colloidal nanoparticles and a 0.2 wt% suspension of anionic silica colloidal nanoparticles. This study demonstrated that building layers by spraying is more efficient for achieving a homogeneous coverage, as well as suppressing the dripping of the polyester fabric [124].

**Figure 5:**
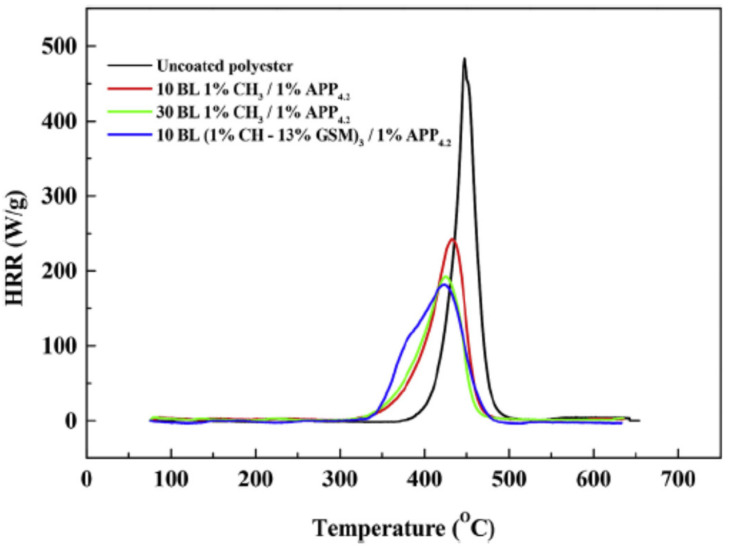
MCC curves of heat release rates as a function of temperature for uncoated and coated polyester [126] Reproduced with permission from Jordanov, I.; Magovac, E.; Fahami, A.; Lazar, S.; Kolibaba, T.; Smith, R.J.; Bischof, S.; Grunlan, J.C. Flame, Polym. Degrad. Stab.; published by Elsevier Ltd., 2019.

Apaydin et al. combined cationic PAH, anionic sodium polyphosphate PSP, a flame retardant and a negatively charged suspension of titanium dioxide (TiO_2_) in 5, 10 and 15 QLs of PAH/PSP/PAH/TiO_2_. However, even at 15 QLs, the pHRR decreased only by 14%, which means that the treatment had little influence on flammability [125]. PAH has been used as a cationic polyelectrolyte rich in nitrogen for the preparation of hollow microcapsules for biomedical drug delivery [132]. Jordanov et al. showed that, by adding low-molecular-weight FR compounds into a 1 wt% cationic CH network, in combination with a 1 wt% anionic APP suspension, it was possible to reduce the number of bilayers needed to pass the VFT (from 30 BLs to 10 BLs). As low-molecular-weight FR additives, nitrogen and nitrogen/sulfur-based derivatives such as 13 wt% guanidine sulfamate (GSM), 13 wt% U, or 13 wt% thiourea (THU) were used in the cationic CH solution. The sample coated with 10 BLs of CH–GSM/APP showed the same self-extinguishing properties as polyester coated with 30 BLs of CH/APP. Moreover, the 10 BLs CH–GSM/APP coating reduced the pHRR by 61.7% relative to uncoated polyester, as shown in Figure 5 [126].

**Figure 6:**
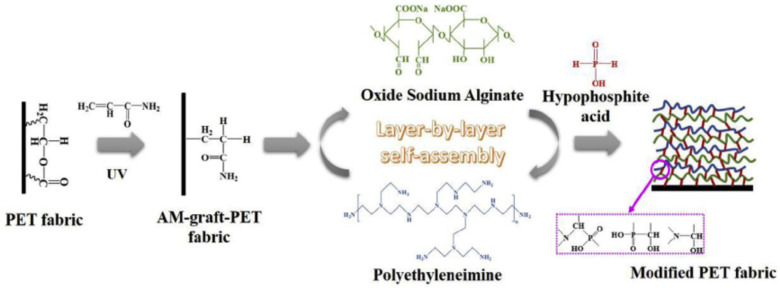
Schematic of LbL deposition of pre-treated MA-graft-polyester (PET) fabric coated with 15 BLs of OSA/PEI and post-treated with HA cross-linking [130] Reproduced with permission from Pan, Y.; Liu, L.; Song, L.; Hu, Y.; Wang, W.; Zhao, H., Polym. Degrad. Stab.; published by Elsevier Ltd., 2019.

Other authors used nitrogen-rich high-molecular-weight cationic PEI or BPEI for coating polyester. Wattanatanom et al. studied the influence of polyelectrolyte concentrations, as well as the number of layers, on the flammability, break strength and stiffness of LbL treated polyester fabric, including wash resistance of FR coating. Another study used a 0.5 wt% cationic BPEI solution and a 5, 7 and 10 wt% anionic APP suspension to reduce flammability and anti-dripping properties. The fabric was first padded in BPEI solution, dried at 80 °C and then padded in APP solution and dried at 110 °C to deposit three, five and seven BLs. Increasing the number of bilayers (three, five and seven BLs) or the concentration of the solution (5, 7 and 10 wt%) improves flame retardancy and anti-dripping of polyester by decreasing after-flame time of coated fabric and self-extinguishing the flame [127]. In a second study with the same formulations, they showed that increasing the concentration of APP, as well as the number of layers, led to an increase in the break strength and stiffness of the fabric, indicating that FR finishing via LBL deposition did not degrade the strength. The formulation of 10 wt% APP at seven BLs showed wash durability of the FR coating for one washing cycle [128]. Carosio et al. investigated how adding salt into solutions influenced the layers, improving the FR properties with the same number of BLs. The authors used 0.1 wt% cationic BPEI as a primer layer to functionalize polyester. The fabric was then immersed into a 0.7 wt% anionic MMT suspension, rinsed in deionized water and then immersed into cationic 1 wt% octapropylammonium polyhedral oligomeric silsesquioxane (OAPOSS) to deposit five BLs. Adding 0.10 M sodium chloride (NaCl) into both the cationic and anionic solutions modified the ionic strength of the systems, which resulted in thicker and more homogeneous coatings. A thicker coating decreased flame spread rate in horizontal flammability tests relative to fabric with the same number of BLs without added NaCl and the fabric showed no melt-dripping. The FR coating showed the same performance after a 1 h washing at 70 °C [129]. Pan et al. alkali hydrolyzed polyester (PET) fabric, UV-grafted it with commercial thickening agent acrylamide (AM) and benzophenone and coated this pre-treated fabric with 5, 10 and 15 BLs of a 0.5 wt% cationic PEI solution and a 0.3 wt% anionic oxide sodium alginate (OSA) solution, a natural polysaccharide found in brown algae. After LbL treatment, the fabrics were immersed into 10 wt% hypophosphorus acid for crosslinking, as shown in Figure 6. Fabric treated with 15 BLs did not show any melt-dripping in horizontal flammability tests and the fire self-extinguished. The pHRR and THR values decreased by 44% and 29.4% relative to untreated fabric and the FR treatment was durable for 12 laundering cycles [130].

The influence of dipping time in polyelectrolyte solution on the flammability of polyester fabric was investigated by Jiang et al. One trilayer (TL) was built by immersing the fabric into a 5 wt% cationic sol solution of flexible polysiloxane (SSP) prepared by sol–gel from methyltriethoxysilane (MTES), isopropanol (IPA) and hydroxy-terminated polydimethylsiloxane (PDMSOH) and a 10 wt% anionic PA solution. One TL consisted of SSP/PA/SSP. The dipping time was set to 0, 5, 10, 15 and 20 min. The study showed that the FR properties of the fabric improved with soaking time, so 20 min of soaking exhibited self-extinguishing properties of polyester fabrics during VFTs, with a 65% reduction in pHRR in comparison with untreated polyester. The FR effect of this fabric was durable up to 45 washing cycles [131]. A summation of the polyelectrolytes used to achieve FR of polyester fabrics is provided in Table 3.

Studies dedicated to polyester flame retardancy by means of LbL deposition show that compounds consisting of nitrogen, phosphorus, sulfur, or inorganic particles reduced the pHRR values by 65% and the THR values by 29%, relative to untreated polyester. By combining different compounds, varying their concentration and applying varying number of bilayers, polyester fabric exhibited no melt dripping and a self-extinguishing behavior. The same effect could be achieved by increasing the immersion time of the fabric in a polyelectrolyte solution [131]. Generally, LbL deposition did not degrade the strength of the polyester fabric [128]. Studies showed that the wash durability of FR treatment depended almost exclusively on the creation of covalent bonds between layers, which could be improved by post-curing with adequate crosslinking agent [131].

### 4.4. Layer-by-Layer Deposition to Reduce Flammability of Polyamide Textiles

The compounds used for FR LbL deposition of polyamide are similar to those applied to cotton and polyester. According to the literature, polyamide is mainly treated with cationic polymers, such as PAH, CH and PEI, as a primer layer or one of the polyelectrolyte pairs [125,133,134,135,136]. As a pre-treatment, chemical grafting with PAA as well as enzymatic modification have been reported [137,138]. Apaydin et al. experimented with a 1 mg/mL cationic PAH solution and a 1 wt% anionic MMT suspension to deposit 5, 10 and 20 BLs on PA6. Cone calorimetry revealed that 20 BLs reduced the pHRR values by more than 60% [133]. These same researchers deposited cationic PAH with anionic PSP to build 5, 10, 15 and 40 BLs on PA6.6. TGA showed that the amount of residue increased for 20 and 40 BLs, while the cone calorimeter data showed a significant decrease in pHRR (up to 36%) for all coated fabrics [134]. The same group of authors combined cationic PAH, anionic PSP and an anionic suspension of titanium dioxide (TiO_2_) to deposit 5, 10 and 15 QLs of PAH/PSP/PAH/TiO_2_ (Section 4.3). Cone calorimetry showed that the coating reduced pHRR by 26% for PA6.6 fabric treated with 15 QLs, but the presence of TiO_2_ did not significantly improve the FR performance relative to the formulation without TiO_2_ [125]. Kumar Kundu et al. deposited 5, 10 and 15 QLs of cationic CH, anionic PA and anionic oxide sodium alginate (OSA) on PA6.6. The aldehyde groups in OSA formed strong covalent bonds with CH and it could be used in LbL deposition as a cross linker. In the VFT, 10 and 15 QL coatings stopped the melt-dripping of PA6.6, with LOI values of ~22%. Cone calorimetry showed that a maximum reduction (24%) in the pHRR was achieved with five QL deposition [135]. In 2018, the same group of authors treated PA6.6 with 5 and 10 BLs of a 1 wt% cationic CH solution and a 2 wt% anionic PA solution to build 5 and 10 BLs. The fabrics were further impregnated in 1 and 5 wt% Na-tetraborate decahydrate solutions and cured at 90 °C. All the treated fabric samples could stop melt dripping in VFTs and pHRR values were lowered compared with the control. In terms of FR performance, the best results were with fabrics treated with 10 BLs (a 31% reduction in pHRR relative to untreated fabric). This coating remained durable up to five washing cycles for PA6.6 impregnated with borate [136]. In 2020, they deposited a 1 wt% cationic CH solution and an anionic solution of 1 wt% phosphorylated chitosan (PCH) and 0.25 wt% poly-acrylate sodium (PAS) onto PA6.6 via “one pot” and LbL deposition to compare the efficiency of these two methods in the reduction in the flammability of PA6.6. Fabric treated via “one pot” for 5 and 10 min was then UV-grafted. Layered fabric was immersed first into cationic CH, washed with DI and then immersed into anionic (PCH-PAS), forming 5 and 10 BLs, then either UV-cured (5 and 10 BLs) or thermally crosslinked (10 BLs). The results indicated that the UV-grafted fabric treated with 10 BLs, with a higher weight gain%, exhibited the highest LOI value of 23% and a 25% reduction in pHRR relative to untreated fabric. However, only the thermally cross-linked PA6.6 treated with 10 BLs retained the FR performance after 5 washing cycles [139]. One-pot synthesis is an expression denoting that all the reactants are subjected to successive chemical reactions in just one reactor, thus saving time and resources and improving the efficiency of a chemical reaction [140].

**Figure 7:**
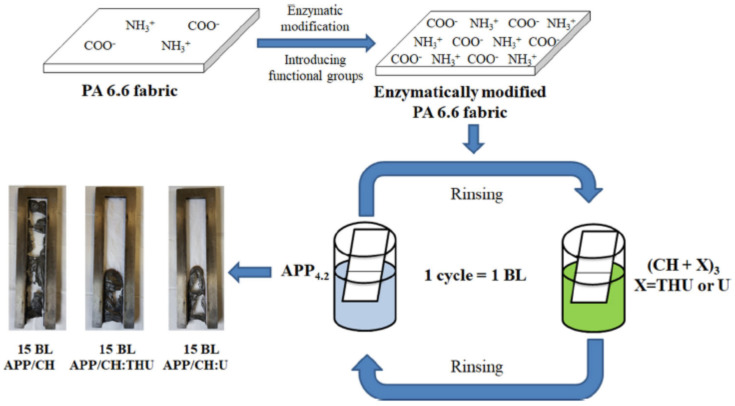
LbL deposition scheme of APP/CH+X on enzymatically modified PA6.6 fabric [138] Reproduced with permission from Jordanov, I.; Kolibaba, T.J.; Lazar, S.; Magovac, E.; Bischof, S.; Grunlan, J.C., J. Mater. Sci.; published by Springer Nature Switzerland AG., 2020.

Ziaur Rahman et al. investigated the influence of pre-treatment and post-treatment on the thermal properties of PA6.6 deposited with two and five BLs of a cationic CH solution with added ME and U and an anionic PA solution. The fabrics were first chemically grafted with PAA in a solution of benzene and dibenzoyl peroxide (BPO). The fabrics were then dipped into cationic PEI, dried at 70 °C and then dipped into a polyacrylic acid–co-maleic acid solution (PAACM) and dried. After LbL treatment, the fabrics were impregnated in a cationic CH and graphene oxide (GO) solution through a pad–dry–cure process. Despite excellent hydrophilic properties achieved by adding GO, none of the treated fabrics passed VFTs either before or after washing [137]. In 2020, Jordanov et al. successfully deposited 15–25 BLs of a 1 wt% anionic APP suspension and a 1 wt% cationic CH solution, with added low-molecular-weight compounds 20 wt% THU or U, onto the enzymatically modified surface of PA6.6 fabrics. The process is schematically shown in Figure 7. By adding low-molecular-weight FR compounds into the CH network, the number of BLs passing the HFT was reduced from 25 BLs of APP/CH-U to 15 BLs, while the pHRR was reduced by 35% relative to untreated fabric [138]. A summation of polyelectrolytes used to achieve FR of polyamide fabrics is provided in Table 4.

There are relatively few studies about LbL deposition of FR compounds on polyamide fabrics, showing reductions in pHRR from 24 to 60% relative to untreated fabric. The results of VFTs and HFTs showed decreased melt dripping. By varying different coating parameters (FR compounds, concentration and number of bilayers), polyamide fabrics could be self-extinguishing. Wash durability (up to five washing cycles) of FR LbL treatment could be achieved by low-temperature thermal curing [139].

### 4.5. Layer-by-Layer Deposition to Reduce Flammability of Cotton/Polyester and Cotton/Polyamide Blends

There are very few studies dealing with LbL deposition of cotton/polyester blends to obtain FR properties. Carosio et al. treated cotton/polyester fabrics with a quadlayer (QL) combination of PDAC/PAA/PDAC/APP. The resulting coating limited the flammability of the fabric by suppressing the afterglow and melt dripping, as well as lowering heat release during combustion (Section 4.1) [107]. Wattanatanom et al. studied the influence of polyelectrolyte concentration, as well as the number of layers, on the flammability of cotton/polyester blends. By using a cationic BPEI solution and a 5, 7 and 10 wt% anionic APP suspension, the flammability and anti-dripping properties of the fabric were reduced with three, five and seven BLs (Section 4.3). The study showed that the increase in the number of bilayers or the concentration of the solution improved the flame retardancy and anti-dripping of blends by decreasing the after-flame time of coated fabrics and self-extinguishing the flame [127]. Alongi et al. investigated whether different orders of layers with the same compounds and same concentration had any influence on the reduction in flammability and anti-dripping behavior of cotton/polyester blends. They used a 0.2 wt% cationic suspension of alumina-coated silica nanoparticles, 0.2 wt% cationic CH solution, 0.2 wt% anionic suspension of silica nanoparticles and 0.2 wt% anionic APP suspension to deposit 5 and 10 silica+/silica-/CH/APP QLs and 5 + 5 and 10 + 10 (CH/APP + silica^+^/silica^−^) BLs on fabric blends. The coated fabric did not pass VFTs, proving that only the thickness of the coating and weight gain had an influence on FR properties [141]. In 2012, Carosio et al. coated two blend fabrics, one with a 0.2 wt% cationic CH solution and a 0.2 wt% anionic APP suspension, depositing 5, 10 and 20 BLs, and a second fabric with a 0.2 wt% cationic suspension of alumina-coated silica nanoparticles with 0.2 wt% APP. Despite the fact that both FR coatings suppressed the afterglow phenomenon, leaving a remarkable residue after combustion, none of the fabrics passed VFTs [142]. In a previous study by Carosio et al. already mentioned in Section 4.1, the burning rate of cotton/polyester blends was successfully reduced in HFTs relative to untreated fabric by combining PDAC/PAA/PDAC/APP in 1, 5 and 10 QLs [107].

**Figure 8:**
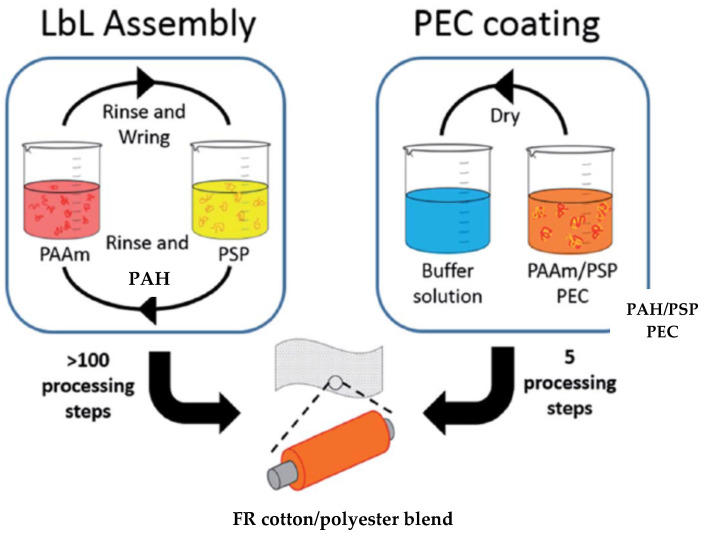
LbL and “one pot” deposition scheme of cotton/polyester fabric with PAH and PSP [143] Reproduced with permission from Haile, M.; Leistner, M.; Sarwar, O.; Toler, C.M.; Henderson, R.; Grunlan, J.C., RSC Adv.; published by Royal Society of Chemistry, 2016.

In 2016, Haile et al. compared the efficiency of two types of coating, LbL and “one pot” deposition, in extinguishing flames during VFTs, as well as the wash durability to home laundering of FR finishes. Blend fabrics were coated by means of LbL deposition with a 1 wt% cationic PAH solution and a 2 wt% anionic PSP suspension (20, 25 and 30 BLs) and by a “one pot” deposition of a water-soluble polyelectrolyte complex suspension (PEC) consisting of three different wt% concentrations (low, medium and high) of PAH and PSP. The LbL-coated fabric was dried at 70 °C, while “one pot” fabrics were dried and then immersed into a buffer solution consisting of citric acid and sodium citrate at pH 4 for 5 min, as shown in Figure 8. In the acidic environment, PAH and PSP formed an insoluble complex, durable up to five laundry cycles. VFTs showed that the highly concentrated “one pot”-coated cotton/polyester fabric with 17.9% weight gain was able self-extinguish, while the MCC data showed a reduction in pHRR of 78% and 31% for cotton and polyester, respectively. The coating process was reduced from more than 100 processing steps to only 5 [143].

**Figure 9:**
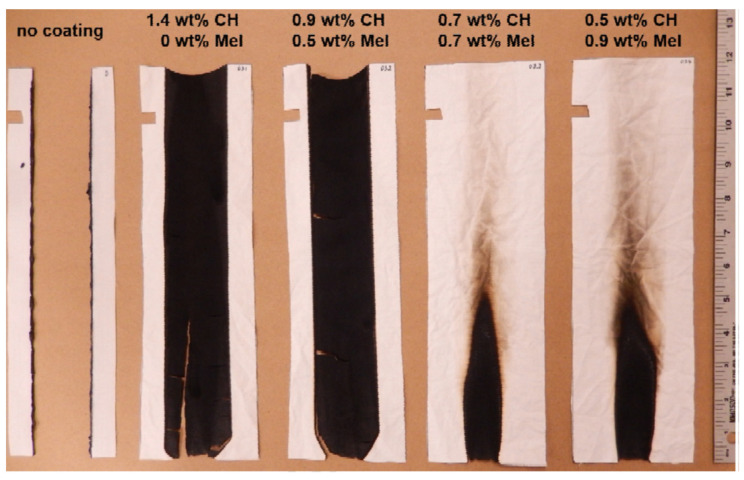
Results of VFT of CH/PSP coating on fabric with melamine addition [144] Reproduced with permission from Leistner, M.; Abu-Odeh, A.A.; Rohmer, S.C.; Grunlan, J.C., Carbohydr. Polym.; published by Elsevier Ltd., 2015.

Leistner et al. investigated the influence of low-molecular-weight additives (e.g., melamine) into the cationic CH network for effective FR properties of cotton/polyester blends coated with a 1.4 w% cationic CH solution and a 2 wt% anionic PSP solution. In this study, the concentration of the cationic solution was held constant at 1.4 wt%, but the concentrations of single components in the cation solution (CH and ME) were different, as shown in Figure 9. The number of bilayers required for a 12.5 wt% coating was 8 BLs for 1.4 wt% CH and 15 BLs for 0.5 wt% and 0.9 wt% melamine, where the latter showed the best result in VFTs, with a char length of 4.5 in and char residue of 93% after performing a combustion calorimeter test [144].

Liu et al. also used ME as a low-molecular-weight additive in a cationic PAH solution. Cotton/polyester fabric was first pretreated with a 1 wt% anionic PAA solution for a better adhesion of LbL layers. The fabric was then immersed into a 1 wt% cationic PAH or PAH–ME solution and a 1 wt% anionic APP suspension, forming 10 BLs. Fabric treated with PAH–ME/APP self-extinguished, with a char length of 11.3 cm in VFTs and with an LOI value of 28.4%. The pHRR was reduced by 34.4%, with a 9 wt% coating [145]. The same group of authors pre-treated cotton/polyester fabric with 0.1 wt% anionic PAA and then immersed it into a 0.5 wt% cationic BPEI solution and a 1 wt% or 2 wt% anionic hypophosphorous acid-modified chitosan (PCH) solution, depositing 10 and 20 BLs. During HFTs, the flame was completely extinguished for the sample coated with 20 BLs of 2 wt% PCH [146]. By depositing alkali-hydrolyzed cotton/polyester blends with a 0.5 wt% cationic PEI solution and a 0.3 wt% anionic OSA solution, thus forming 5 and 10 BLs, and then soaking coated fabrics into a 10 wt% HA solution for cross-linking, it was possible to achieve self-extinguishing in HFTs with FR coating durable through 12 home laundry cycles [147]. Wang et al. combined a 1 wt% cationic γ-paperazinylproplymethyldimethoxy silane (GP-108) solution with a 1 wt% anionic APP solution to build up 5, 10 and 15 BLs. Fabric coated with 15 BLs achieved self-extinguishing in VFTs and showed a strong decrease in heat release during cone calorimetry tests [148].

The number of studies on LbL deposition to reduce the flammability of cotton/polyamide blends is very limited. Narkhede at al. first pre-treated these blends by immersing them into a pH 2 solution for cationization. The cationized fabric was then deposited with 5, 10, 15 and 20 BLs by dipping. For the anionic polyelectrolyte, a 2 wt% PSP solution was used and, for the polycationic, three different cationic polysiloxane compounds were used, namely, 6.8 wt% (trimethylammonium methyl phenythyl)-methyl siloxane and dimethyl siloxane copolymer chloride salt (QMS-435) solution, 4 wt% aminoethylaminopropyl silsesquioxane–methylsilsesquioxane copolymer oligomer (WSA-7021) solution and 4 wt% aminopropyl silesquioxane oligomers (WSA-9911) solution. Only fabrics coated with 20 BLs of WSA-7021 and WSA-9911 passed VFTs [149]. A summation of polyelectrolytes used to achieve FR of blend fabrics is provided in Table 5.

The reduced flammability of cotton blends can be easily achieved with a wide range of chemical compounds containing nitrogen, phosphorus, sulfur and inorganic compounds, as summarized in Table 5. As a pre-treatment, various primer layer chemicals have been used, such as BPEI or PAA, or the cotton blends have been treated with acid/alkali hydrolysis to achieve more functional groups on the fiber surface. By means of FR LbL deposition, the pHRR values were reduced by 78% and 31% for the cotton and polyester. Wash durability of FR LbL treated blends could be achieved by low-temperature thermal curing (up to 12 washing cycles) [147]. However, the role of each FR chemical compounds in LbL recipes and their mode of action on suppression of flames on cotton blends require further analyses, but the generally accepted opinion is that these compounds act as passive barriers and/or intumescent of known modes of actions.

## 5. Conclusions

LbL deposition has long been considered one of the green alternatives for current commercially available finishing technologies to impart flame retardancy to the most widely used fibers globally—cotton, polyester, polyamide and their blends. It is possible to use almost any compound consisting of nitrogen, phosphorus, sulfur, or metal in the form of small organic molecules (U, THU and MEL), synthetic long-chain macromolecules (APTES, PDAC, PAA, APP, BPEI, PAH, PSP and SSP), biomacromolecules (DNA, egg white protein, PA, CS, SA, CH and its derivates), or inorganic colloids, such as metal, metal oxides and clays. By means of FR LbL deposition, the pHRR values of cotton can be reduced up to 97% and the THR values by 93% relative to untreated fabric, with minimal influence on break strength. With the same technique, the pHRR values of treated polyester can be reduced up to 65% and the THR values by 29% relative to untreated fabric. Additional benefits are the suppression of melt dripping and self-extinguishing behavior. Generally, LbL deposition does not degrade the strength of the polyester. Limited studies regarding LbL deposition of FR compounds on polyamide fabric showed the reduction in pHRR within the range 24–60%. The results of VFT and HFT showed improvements and a decrease in melt dripping. The pHRR values of cotton and polyester in blends were reduced by 78% and 31%, respectively. Comparable to commercially available and industrially feasible technologies of FR finishing, one of the advantages of the LbL technique is the use of deionized water as a solvent for a very low concentration of polyelectrolytes (up to 10 wt%).

Since the layers are bound by weak electrostatic and hydrogen bonds, sensitive to environmental conditions (pH, dipping time, electrolyte concentration and purity), flame-retardant (and multifunctional) LbL finishing is not durable to conventional laundering. This drawback can be partially eliminated by low-temperature thermal curing to form covalent bonds between layers. Technological drawbacks of LbL deposition are the use of high amounts of water as well as time to achieve the desired number of layers sufficient for effective reduction in flammability. In the case of using eco-friendly chemical compounds, such as DNA, PA, CH, or egg proteins, one should be aware of their high costs. LbL deposition of FR compounds onto the textile materials is a promising alternative to overcome the negative drawbacks of current commercially available technologies, via the elimination of free formaldehyde during the product life cycle or the use of eco-friendly chemicals from renewable sources.

## Figures and Tables

**Table 1:** Polyelectrolytes used to achieve FR of cotton.

Pre-Treatment	Recipe	Number of Layers	Literature
	BPEI^+^/MMT^−^	5, 20 BLs	[85]
	CS^+^/MMT^−^	5, 10, 20 BLs	[93]
	APTES^+^/PA^−^	5, 10, 15 BLs	[99]
	(PEI+SiO_2_)^+^/PA^−^	2, 4, 7 BLs	[94]
	(PEI+SiO_2_)^+^/PPA^−^	1 BLs	[101]
	(PEI+ME)^+^/PA^−^	2, 4 BLs	[90]
	APTES^+^/PA^−^/CH^+^/PA^−^	5, 10, 15 QLs	[86]
Primer layer APTES^+^	APP^−^/CH^+^	2, 3, 6 BLs	[87]
	CH^+^/(SMF+PA)^−^	5, 10 BLs	[91]
	PEI^+^/PA^−^	8 BLs	[88]
Primer layer BPEI^+^	PA^−^/PVAm^+^	5, 10, 15 BLs	[89]
Primer layer BPEI^+^	PA^−^/(CH+U)^+^	8, 10, 12, 15 BLs	[92]
	Egg white protein^+^/PA^−^	1 BLs	[105]
	PDAC^+^/PAA^−^/PDAC^+^/APP^−^	1, 5, 10 QLs	[107]
	PDAC^+^/DNA^−^	5, 10 BLs	[108]
	PVA^+^/(GNP+PSS)^–^	10 BLs	[95]
	PEI^+^/SA^−^	10 BLs	[109]
	PEI^+^/HACH^−^	5, 10 BLs	[111]
	CH^+^/APP^−^(CH+AMM)^+^/APP^−^	5, 10, 15 BLs	[112]
Primer layer PEI^+^	PSP^−^/CH^+^	5, 10, 15 BLs	[113]

**Table 2:** Polyelectrolytes used to achieve FR and other multifunctional properties of cotton.

Pre-Treatments	Recipe	Number of Layers	Properties	Literature
Primer layer PEI^+^	GO^−^/CA^+^	1, 5, 10 BLs	FR, antistatic	[115]
Primer layer GO^−^	APTES^+^/SA^−^/APP^−^/GO^−^	10 QLs on 2 sides of fabric	FR, antistatic, antibacterial	[116]
Primer layer PEI^+^	PA^−^/PCQS^+^, immersion into NaClO	10, 20, 30 BLs	FR, antibacterial	[117]
	CH^+^/AP^−^	1 BL	FR, antibacterial	[118]
Primer layer BPEI^+^	PA^−^/(CH + U)^+^, immersion into CuSO_4_	8, 10, 12 BLs	FR, antibacterial	[104]
	(PEI + SiO_2_)^+^/PPA^−^, immersion into repellent finish	1 BL	FR, hydrophobic	[101]
	(PEI + ME)^+^/PA^−^, immersion into chloroform solution	4 BLs	FR, hydrophobic	[90]
	BPEI^+^/APP^−^	1BL	FR, hydrophobic	[119]
	PDDA^+^/BNNS^−^	16 BLs	FR, hydrophobic	[120]
	(CH + citric acid + SHP)/(SLS + BA)	1, 2, 3 BLs	FR, antibacterial, UV-protective, wrinkle resistant	[121]
	(BPEI^+^/CNTs)_10_/ BPEI^+^/APP^−^ (BPEI^+^/CNTs)_10_/BPEI^+^/APP^−^/PDMS	10 BLs+1 BL 10 BLs+1 TL	FR, conductive FR, conductive, hydrophobic	[122]
	PEI^+^/PA^−^	8 BLs	FR, electromagnetic interference shielding	[88]

**Table 3:** Cationic/anionic compounds used to achieve FR of polyester fabrics.

Pre-Treatment	Recipe	Number of Layers	Literature
	PDAC^+^/PAA^−^/PDAC^+^/APP^−^	1, 5, 10 QLs	[107]
	PDAC^+^/ZrP^−^ SiO_2_^+^/ZrP^−^ POSS^®+^/ZrP^−^	5, 10 BLs	[123]
	Ludox CL^+^/Ludox SM30^−^	5 BLs	[124]
	PAH^+^/PSP^−^/PAH^+^/TiO_2_^−^	5, 10, 15 QLs	[125]
	CH^+^/APP- (CH+GSM)^+^/APP^−^ (CH+U)^+^/APP^−^ (CH+THU)^+^/APP^−^	10, 25, 30 BLs	[126]
	BPEI^+^/APP^−^	3, 5, 7 BLs	[127,128]
Primer layer BPEI^+^	MMT^−^/OAPOSS^+^	5 BLs	[129]
1. Alkali hydrolysis 2. UV-grafting with AM and benzophenone	PEI^+^/OSA^−^	5, 10, 15 BLs	[130]
	SSP^+^/PA^−^/SSP^+^	1 TL	[131]

**Table 4:** Polyelectrolytes used to achieve FR of polyamide fabric.

Pre-Treatment	Recipe	Number of Layers	Literature
	PAH^+^/PSP^−^/PAH^+^/TiO_2_^−^	5, 10, 15 QLs	[125]
	PAH^+^/MMT^−^	5, 10, 20 BLs	[133]
	PAH^+^/PSP^−^	5, 10, 15, 40 BLs	[134]
	CH^+^/PA^−^/CH^+^/OSA^−^	5, 10, 15 QLs	[135]
	CH^+^/PA^−^	5, 10 BLs	[136]
	CH^+^/(PCH+PAS)^−^	1BL	[139]
1. Chemical grafting with PAA in solution of benzene and BPO 2. PEI^+^/PAACM^−^, dipping, drying	(CH+ME+U)^+^/PA^−^	2, 5 BLs	[137]
Enzymatic modification with protease from *Bacillus licheniformis*	APP^−^/CH^+^ APP^−^/(CH+THU)^+^ APP^−^/(CH+U)^+^	15, 30 BLs 10, 15 BLs 10, 15 BLs	[138]

**Table 5:** Polyelectrolytes used to achieve FR of cotton/polyester and cotton/polyamide blends.

Pre-Treatments	Recipes	Number of Layers	Literature
Cotton/polyester			
	PDAC^+^/PAA^−^/PDAC^+^/APP^−^	1, 5, 10 QLs	[107]
	BPEI^+^/APP^−^	3, 5, 7 BLs	[127]
	alumina-coated silica nanoparticles^+^/silica nanoparticles^−^/CH^+^/APP^−^ CH^+^/APP^−^ +alumina-coated silica nanoparticles^+^/silica nanoparticles^−^	5, 10 QLs 5 + 5 BLs 10 + 10 BLs	[141]
	CH^+^/APP^−^ alumina-coated silica nanoparticles^+^/APP^−^	5, 10, 20 BLs	[142]
	PAH^+^/PSP^−^	20, 25, 30 BLs	[143]
	CH^+^/PSP^−^ (CH+ME)^+^/PSP^−^	8 BLs 10, 15 BLs	[144]
Primer layer PAA^−^	PAH^+^/APP^−^ (PAH+ME)^+^/APP^−^	10 BLs 10 BLs	[145]
Primer layer PAA^−^	BPEI^+^/PCH^−^	10, 20 BLs	[146]
Alkali hydrolysis	OSA^−^/PEI^+^	5, 10 BLs	[147]
	GP-108^+^/APP^−^	5, 10, 15 BLs	[148]
Cotton/polyamide			
	PSP^−^/QMS-435^+^ PSP^−^/WSA-7021^+^ PSP^−^/WSA-9911^+^	5, 10, 15, 20 BLs	[149]

## Data Availability

Data is contained within the article.

## Abbreviations

AgNW	silver nanowires
AM	acrylamide
AMM	p—aminobenzene sulfonic acid modified melamine
AP	ammonium phytate
APP	ammonium polyphosphate
APTES	3-aminopropyl triethoxysilane
BA	boric acid
BaCl_2_	barium chloride
BL	bilayer
BNNS	boron nitride nanosheets
BOD	biochemical oxygen demand
BPEI	branched polyethyleneimine
BPO	dibenzoyl peroxide
BTCA	butyl tetracarboxylic acid
C_4_H_6_O_4_Co·4H_2_O	cobalt acetate tetrahydrate
C_4_H_6_O_4_Ni·4H_2_O	nickel acetate tetrahydrate
CA	anionic caprolactam modified casein composite emulsion
CH	chitosan
CNT	carbon nanotubes
CO	carbon monoxide
CO_2_	carbon dioxide
COD	chemical oxygen demand
CS	cationic starch
DI	deionized water
DMT	dimethyl terephthalate
DNA	deoxyribonucleic acid
FR	flame retardant
F-SiO_2_	fluorinated silica
GNP	graphene nanoplatelets
GO	graphene oxide
GP-108	γ-paperazinylproplymethyldimethoxy silane
GSM	guanidine sulfamate
HA	hypophosphorus acid
HACH	hypophosphorous acid-modified chitosan
HBr	hydrogen bromide
HCl	hydrogen chloride
HCN	hydrogen cyanide
HF	hydrogen fluoride
HFT	horizontal flammability test
HT	hydrotalcite
IPA	isopropanol
LbL	layer-by-layer
LOI	limiting oxygen index
Ludox CL	alumina coated silica
Ludox SM30	silica
MCC	microscale combustion calorimeter
ME	melamine
MMT	sodium montmorillonite
MTES	methyltriethoxysilane
NaBH_4_	sodium borohydride
NaCl	sodium chloride
NaClO	sodium hypochlorite
NH_3_	ammonia
N-MDMPA	N-methylol dimethylphosphonopropionamide
NO_x_	nitrogen oxide
OAPOSS	octapropylammonium polyhedral oligomeric silsesquioxane
OSA	oxide sodium alginate
PA	phytic acid
PAA	poly (acrylic acid)
PAACM	polyacrylic acid-co-maleic acid solution
PAH	poly (allylamine hydrochloride)
PAS	polyacrylate sodium
PCH	phosphorylated chitosan
PCQS	poly[3-(5,5-cyanuricacidpropyl)- siloxane-co-trimethylammoniumpropylsiloxane chloride]
PDAC	poly (diallyl dimethylammonium chloride)
PDDA	poly dimethyl diallyl ammonium chloride
PDMS	polydimethylsiloxane
PDMSOH	hydroxy-terminated polydimethylsilo
PEC	stable soluble polyelectrolyte complex (PSP and PAH)
PEI	polyethyleneimine
pHRR	peak heat release rate
POSS^®^	polyhedral oligomeric silsesquioxane cage molecule carrying eight n-propylammonium chloride groups
PPA	polyphosphoric acid
PSP	sodium polyphosphate
PSS	poly (4-styrenesulfonic acid);
PVA	polyvinyl alcohol
PVAm	polyvinyl amine
QL	quadlayer
QMS-435	(trimethylammonium methyl phenylethyl)-methyl siloxane and dimethyl siloxane copolymer chloride salt
SA	sodium alginate
SHP	Sodium Hypophosphite
SiO_2_	alumina-coated silica nanoparticles
SLS	sodium lignin sulphonate
SMF	sulfonated melamine-formaldehyde
SSP	flexible polysiloxane prepared by sol-gel from methyltriethoxysilane (MTES), isopropanol (IPA) and hydroxy-terminated polydimethylsiloxane (PDMSOH)
TA	therephthalic acid
T_g_	glass transition temperature
THAM	tris(hydroxymethyl)aminomethane
THPX	tetrakis(hydroxymethyl) phosphonium salt
THR	total heat release rate
THU	thiourea
TiO_2_	titanium dioxide
TL	trilayer
T_p_	melting point
T_p_	thermal decomposition
U	urea
VFT	vertical flammability test
WSA-7021	aminoethylaminopropyl silsesquioxane–methylsilsesquioxane copolymer oligomer
WSA-9911	aminopropyl silesquioxane oligomers
ZrP	α-zirconium phosphate

## References

[1] Shishoo R., Shishoo R. (2012). Introduction: Trends in the global textile industry. The Global Textile and Clothing Industry: Technological Advances and Future Challenges.

[2] Environmental Impact of Textile and Clothes Industry. https://www.europarl.europa.eu/RegData/etudes/BRIE/2019/633143/EPRS_BRI(2019)633143_EN.pdf.

[3] World Fire Statistics. https://www.ctif.org/sites/default/files/2020–06/CTIF_Report25.pdf.

[4] Global Flame Retardants Market by Country, Product and Market. https://www.freedoniagroup.com/industry-study/global-flame-retardants-market-by-country-product-and-market-6th-edition-3499.htm.

[5] Magovac E., Bischof S. (2015). Non-Halogen FR Treatment of Cellulosic Textiles. Tekstil.

[6] Liang S., Neisius N.M., Gaan S. (2013). Recent Developments in Flame Retardant Polymeric Coatings. Prog. Org. Coat..

[7] Lewin M., Weil E.D., Horrocks A.R., Price D. (2001). Mechanisms and Modes of Action in Flame Retardancy of Polymers. Fire Retardant Materials.

[8] Beninate J.V., Boylston E.K., George L., Drake J., Reeves W.A. (2016). Conventional Pad-Dry-Cure Process for Durable- Flame and Wrinkle Resistance with Tetrakis (Hydroxymethyl) Phosphonium Hydroxide (THPOH). Text. Res. J..

[9] Tišler T., Zagorc-Končan J. (1997). Comparative Assessment of Toxicity of Phenol, Formaldehyde, and Industrial Wastewater to Aquatic Organisms. Water Air Soil Pollut..

[10] Horrocks A.R. (2011). Flame Retardant Challenges for Textiles and Fibres: New Chemistry versus Innovatory Solutions. Polym. Degrad. Stab..

[11] Kundu C.K., Li Z., Song L., Hu Y. (2020). An Overview of Fire Retardant Treatments for Synthetic Textiles: From Traditional Approaches to Recent Applications. Eur. Polym. J..

[12] Weil E.D., Levchik S.V. (2008). Flame Retardants in Commercial Use or Development for Textiles. J. Fire Sci..

[13] Sundar S., Chakravarty J. (2010). Antimony Toxicity. Int. J. Environ. Res. Public Health.

[14] Alongi J., Carosio F., Malucelli G. (2014). Current Emerging Techniques to Impart Flame Retardancy to Fabrics: An Overview. Polym. Degrad. Stab..

[15] Malucelli G., Yusuf M. (2018). Bio-macromolecules: A new flame retardant finishing strategy for textiles. Handbook of Renewable Materials for Coloration and Finishing.

[16] Lazar S.T., Kolibaba T.J., Grunlan J.C. (2020). Flame-Retardant Surface Treatments. Nat. Rev. Mater..

[17] Iler R.K. (1966). Multilayers of Colloidal Particles. J. Colloid Interface Sci..

[18] Kirkland J.J. (1965). Porous Thin-Layer Modified Glass Bead Supports for Gas Liquid Chromatography. Anal. Chem..

[19] Decher G., Hong J.D., Schmitt J. (1992). Buildup of Ultrathin Multilayer Films by a Self-Assembly Process: III. Consecutively Alternating Adsorption of Anionic and Cationic Polyelectrolytes on Charged Surfaces. Thin Solid Film..

[20] Ariga K., Yamauchi Y., Rydzek G., Ji Q., Yonamine Y., Wu K.C.-W., Hill J.P. (2014). Layer-by-Layer Nanoarchitectonics: Invention, Innovation, and Evolution. Chem. Lett..

[21] Alongi J., Carosio F., Frache A., Malucelli G. (2013). Layer by Layer Coatings Assembled through Dipping, Vertical or Horizontal Spray for Cotton Flame Retardancy. Carbohydr. Polym..

[22] Decher G., Decher G., Schlenoff J.B. (2012). Layer-by-Layer Assembly (Putting Molecules to Work). Multilayer Thin Films: Sequential Assembly of Nanocomposite Materials.

[23] Michel M., Toniazzo V., Ruch D., Ball V. (2012). Deposition Mechanisms in Layer-by-Layer or Step-by-Step Deposition Methods: From Elastic and Impermeable Films to Soft Membranes with Ion Exchange Properties. ISRN Mater. Sci..

[24] Wu G., Zhang X., Decher G., Schlenoff J.B. (2012). Layer-by-Layer Assembly: From Conventional to Unconventional Methods. Multilayer Thin Films: Sequential Assembly of Nanocomposite Materials.

[25] Holder K.M., Smith R.J., Grunlan J.C. (2017). A Review of Flame Retardant Nanocoatings Prepared Using Layer-by-Layer Assembly of Polyelectrolytes. J. Mater. Sci..

[26] Chatterjee K., Tabor J., Ghosh T.K. (2019). Electrically Conductive Coatings for Fiber-Based E-Textiles. Fibers.

[27] Wanasinghe D., Aslani F. (2019). A Review on Recent Advancement of Electromagnetic Interference Shielding Novel Metallic Materials and Processes. Compos. Part B Eng..

[28] Zhu X., Jun Loh X. (2015). Layer-by-Layer Assemblies for Antibacterial Applications. Biomater. Sci..

[29] Li S., Huang J., Chen Z., Chen G., Lai Y. (2017). A Review on Special Wettability Textiles: Theoretical Models, Fabrication Technologies and Multifunctional Applications. J. Mater. Chem. A.

[30] Mateos A.J., Cain A.A., Grunlan J.C. (2014). Large-Scale Continuous Immersion System for Layer-by-Layer Deposition of Flame Retardant and Conductive Nanocoatings on Fabric. Ind. Eng. Chem. Res..

[31] Krogman K.C., Zacharia N.S., Schroeder S., Hammond P.T. (2007). Automated Process for Improved Uniformity and Versatility of Layer-by-Layer Deposition. Langmuir.

[32] Jang W.S., Grunlan J.C. (2005). Robotic Dipping System for Layer-by-Layer Assembly of Multifunctional Thin Films. Rev. Sci. Instrum..

[33] Liu G., Zhao J., Sun Q., Zhang G. (2008). Role of Chain Interpenetration in Layer-by-Layer Deposition of Polyelectrolytes. J. Phys. Chem. B.

[34] Mohammadi M., Salehi A., Branch R.J., Cygan L.J., Besirli C.G., Larson R.G. (2017). Growth Kinetics in Layer-by-Layer Assemblies of Organic Nanoparticles and Polyelectrolytes. ChemPhysChem.

[35] Steitz R., Jaeger W., Klitzing R.V. (2001). Influence of Charge Density and Ionic Strength on the Multilayer Formation of Strong Polyelectrolytes. Langmuir.

[36] Belce Y., Cebeci F.C. (2019). Investigation of PH and Concentration Influence on Layer-by-Layer Self-Assembly for Nickel(II)Phthalocyanine-Tetrasulfonic Acid Tetrasodium Salt Coatings. J. Porphyr. Phthalocyanines.

[37] Yang Y.-H., Malek F.A., Grunlan J.C. (2010). Influence of Deposition Time on Layer-by-Layer Growth of Clay-Based Thin Films. Ind. Eng. Chem. Res..

[38] Gamboa D., Priolo M.A., Ham A., Grunlan J.C. (2010). Note: Influence of Rinsing and Drying Routines on Growth of Multilayer Thin Films Using Automated Deposition System. Rev. Sci. Instrum..

[39] Stawski D., El Nemr A. (2012). Application of the Layer-by-layer Method for Textiles. Textiles, Uses and Production Methods.

[40] Kashiwagi T. (1994). Polymer Combustion and Flammability-Role of the Condensed Phase. Symp. Combust..

[41] Moldoveanu S.C., Moldoveanu S.C. (2019). General Information About Pyrolysis. Pyrolysis of Organic Molecules.

[42] Hull T.R., Law R.J., Bergman Å., Papaspyrides C.D., Kiliaris P. (2014). Environmental drivers for replacement of halogenated flame retardants. Polymer Green Flame Retardants.

[43] Hörold S., Papaspyrides C.D., Kiliaris P. (2014). Phosphorus-based and Intumescent Flame Retardants. Polymer Green Flame Retardants.

[44] Brown S.C., Pritchard G. (1998). Flame retardants: Inorganic oxide and hydroxide systems. Plastics Additives.

[45] Textile Exchange Organization Preferred Fiber & Materials Market Report 2020. https://textileexchange.org/about-us/#annualreports.

[46] Thakur V.K., Thakur V.K. (2015). Grafting of Cellulose-Based Materials: Techniques, Factors, and Applications of the Grafted Products. Cellulose-Based Graft Copolymers.

[47] Horrocks A.R. (1983). An Introduction to the Burning Behaviour of Cellulosic Fibres, A.J. Soc. Dye. Colour..

[48] NIIR Board of Consultants and Engineers (2006). NIIR Board of Consultants and Engineers Polyesters. The Complete Technology Book on Expanded Plastics, Polyurethane, Polyamide and Polyester Fibres.

[49] Chang P.-H., Wilkie C.A. (1989). A Mechanism for Flame Retardation of Poly(Ethylene Terephthalate). J. Appl. Polym. Sci..

[50] Richards A.F., McIntyre J.E. (2004). Nylon fibres. Synthetic Fibres: Nylon, Polyester, Acrylic, Polyolefin.

[51] McKeen L.W., McKeen L.W. (2017). Polyamides (Nylons). Film Properties of Plastics and Elastomers.

[52] NIIR Board of Consultants and Engineers (2006). NIIR Board of Consultants and Engineers Polyamides and Polyimides. The Complete Technology Book on Expanded Plastics, Polyurethane, Polyamide and Polyester Fibres.

[53] Braun E., Levin B.C. (1987). Nylons: A Review of the Literature on Products of Combustion and Toxicity Emil. Fire Mater..

[54] Schaffer M.A., Marchildon E.K., McAuley K.B., Cunningham M.F. (2000). Thermal Nonoxidative Degradation of Nylon 6,6. J. Macromol. Sci. Part C Polym. Rev..

[55] Weil E.D., Levchik S. (2004). Current Practice and Recent Commercial Developments in Flame Retardancy of Polyamides. J. Fire Sci..

[56] Subbulakshmi M.S., Kasturiya N., Bajaj P.H., Agarwal A.K. (2000). Production of Flame-Retardant Nylon 6 and 6.6. J. Macromol. Sci. Polym. Rev..

[57] Muralidhara K.S., Sreenivasan S. (2012). Adaptation of Pyrolytic Conduit of Polyester Cotton Blended Fabric with Flame Retardant Chemical Concentrations. Res. J. Chem. Sci..

[58] Muralidhara K.S., Sreenivasan S. (2010). Thermal Degradation Kinetic Data of Polyester, Cotton and Polyester-Cotton Blended Textile Material. World Appl. Sci. J..

[59] Jarvis C.W., Baker R.H., Lewin M., Atlas S.M., Pearce E.M. (1978). Flammability of Cotton-Polyester Blend Fabrics. Flame Retardant Polymeric Materials.

[60] Chen Q., Zhao T. (2016). The Thermal Decomposition and Heat Release Properties of the Nylon/Cotton, Polyester/Cotton and Nomex/Cotton Blend Fabrics. Text. Res. J..

[61] Cooney J.D.D., Day M., Wiles D.M.M. (1986). A Kinetic Study of the Thermal Degradation of Polyester/Cotton Blends by Thermogravimetry. Thermochim. Acta.

[62] Chen Q., Yang C.Q., Zhao T. (2014). Heat Release Properties and Flammability of the Nylon/Cotton Blend Fabric Treated with a Crosslinkable Organophosphorus Flame Retardant System. J. Anal. Appl. Pyrolysis.

[63] Horrocks A.R., Mittal K.L., Bahners T. (2017). Flame Retardant Textile Finishes. Textile Finishing.

[64] Salaspuro M., Lindros K. (1985). Metabolism and Toxicity of Acetaldehyde. Alcohol Relat. Dis. Gastroenterol..

[65] Dasarathy S., Mookerjee R.P., Rackayova V., Rangroo Thrane V., Vairappan B., Ott P., Rose C.F. (2016). Ammonia Toxicity: From Head to Toe?. Metab. Brain Dis..

[66] Riesch R., Tobler M., Plath M., Riesch R., Tobler M., Plath M. (2015). Hydrogen Sulfide-Toxic Habitats. Extremophile Fishes.

[67] Tanii H., Hashimoto K. (1984). Studies on the Mechanism of Acute Toxicity of Nitriles in Mice. Arch. Toxicol..

[68] Alarie Y. (2002). Toxicity of Fire Smoke. Crit. Rev. Toxicol..

[69] Singh S.P., Soni B., Bajpai S.K., Thakur V.K. (2015). Cellulose-Based Graft Copolymers. Cellulose-Based Graft Copolymers.

[70] Grancaric A.M., Tarbuk A., Pusic T. (2005). Electrokinetic Properties of Textile Fabrics. Color. Technol..

[71] East A.J., McIntyre J.E. (2004). Polyester fibres. Synthetic Fibres: Nylon, Polyester, Acrylic, Polyolefin.

[72] Jordanov I., Mangovska B. (2001). Optimal Parameters of Enzymatic Scouring and Compared with Alkaline Scouring. Tekstil.

[73] Buschle-Diller G., Cavaco-Paulo A., Gübitz G.M. (2003). Substrates and their structure. Textile Processing with Enzymes.

[74] Song J.E., Kim H.R. (2016). Improvement in Nylon Fabrics’ Reactivity via Enzymatic Functionalization. J. Text. Inst..

[75] Silva C., Cavaco-Paulo A.M., Fu J.J., Roshan P. (2015). Enzymatic biofinishes for synthetic textiles. Functional Finishes for Textiles: Improving Comfort, Performance and Protection.

[76] Kisner A., Rainert K.T., Ferrari F., Nau C.T., Barcellos I.O., Pezzin S.H., Andreaus J. (2013). Chemical Functionalization of Polyamide 6.6 Fabrics. React. Funct. Polym..

[77] Shearer A.E., Paik J.S., Hoover D.G., Haynie S.L., Kelley M.J. (2000). Potential of an Antibacterial Ultraviolet-Irradiated Nylon Film. Biotechnol. Bioeng..

[78] Yip J., Chan K., Sin K.M., Lau K.S. (2002). Low Temperature Plasma-Treated Nylon Fabrics. J. Mater. Process. Technol..

[79] Samanta K.K., Basak S., Chattopadhyay S.K., Muthu S.S. (2014). Environment-Friendly Textile Processing Using Plasma and UV Treatment. Roadmap to Sustainable Textiles and Clothing.

[80] Butola B.S., Deopura B.L., Alagirusamy R., Joshi M., Gupta B. (2008). Advances in functional finishes for polyester and polyamide-based textiles. Polyesters and Polyamides.

[81] Tarbuk A., Đorđević D., Flinčec Grgac S., Kodrić M., Magovac E., Čorak I., Zdraveva E., Mijović B. (2020). The Influence of Lipase Surface Modification to Polyester Crystallinity and Absorbility. Proceedings of the 13th International Scientific Professional Symposium Textile Science & Economy.

[82] Jenkins R.O., Cavaco-Paulo A., Gübitz G.M. (2003). Enzymes. Textile Processing with Enzymes.

[83] Silva C., Cavaco-Paulo A., Nierstrasz V.A., Nierstrasz V.A., Cavaco-Paulo A. (2010). Enzymatic hydrolysis and modification of core polymer fibres for textile and other applications. Advances in Textile Biotechnology.

[84] Tabujew I., Peneva K., Samal S., Dubruel P. (2014). Functionalization of Cationic Polymers for Drug Delivery Applications. Cationic Polymers in Regenerative Medicine.

[85] Li Y.-C., Schulz J., Mannen S., Delhom C., Condon B., Chang S., Zammarano M., Grunlan J.C. (2010). Flame Retardant Behavior of Polyelectrolyte-Clay Thin Film Assemblies on Cotton Fabric. ACS Nano.

[86] Liu Y., Wang Q.Q., Jiang Z.M., Zhang C.J., Li Z.F., Chen H.Q., Zhu P. (2018). Effect of Chitosan on the Fire Retardancy and Thermal Degradation Properties of Coated Cotton Fabrics with Sodium Phytate and APTES by LBL Assembly. J. Anal. Appl. Pyrolysis.

[87] Chen H.Q., Xu Y.J., Jiang Z.M., Jin X., Liu Y., Zhang L., Zhang C.J., Yan C. (2020). The Thermal Degradation Property and Flame-Retardant Mechanism of Coated Knitted Cotton Fabric with Chitosan and APP by LBL Assembly. J. Therm. Anal. Calorim..

[88] Zhang Y., Tian W., Liu L., Cheng W., Wang W., Liew K.M., Wang B., Hu Y. (2019). Eco-Friendly Flame Retardant and Electromagnetic Interference Shielding Cotton Fabrics with Multi-Layered Coatings. Chem. Eng. J..

[89] Zilke O., Plohl D., Opwis K., Mayer-Gall T., Gutmann J.S. (2020). A Flame-Retardant Phytic-Acid-Based LbL-Coating for Cotton Using Polyvinylamine. Polymers.

[90] Liu L., Huang Z., Pan Y., Wang X., Song L., Hu Y. (2018). Finishing of Cotton Fabrics by Multi-Layered Coatings to Improve Their Flame Retardancy and Water Repellency. Cellulose.

[91] Pan Y., Zhao H. (2018). A Novel Blowing Agent Polyelectrolyte for Fabricating Intumescent Multilayer Coating That Retards Fire on Cotton Fabric. J. Appl. Polym. Sci..

[92] Magovac E., Jordanov I., Grunlan J.C., Bischof S. (2020). Environmentally-Benign Phytic Acid-Based Multilayer Coating for Flame Retardant Cotton. Materials.

[93] Choi K., Seo S., Kwon H., Kim D., Park Y.T. (2018). Fire Protection Behavior of Layer-by-Layer Assembled Starch–Clay Multilayers on Cotton Fabric. J. Mater. Sci..

[94] Li S., Ding F., Lin X., Li Z., Ren X. (2019). Layer-by-Layer Self-Assembly of Organic-Inorganic Hybrid Intumescent Flame Retardant on Cotton Fabrics. Fibers Polym..

[95] Jang W., Chung I.J., Kim J., Seo S., Park Y.T., Choi K. (2018). Improving Fire Resistance of Cotton Fabric through Layer-by-Layer Assembled Graphene Multilayer Nanocoating. J. Korean Phys. Soc..

[96] Li Y., Ju D., Jiang X., Gao H. (2017). The Application, Neurotoxicity, and Related Mechanism of Cationic Polymers. Neurotoxicity of Nanomaterials and Nanomedicine.

[97] Uddin F. (2008). Clays, Nanoclays, and Montmorillonite Minerals. Metall. Mater. Trans. A.

[98] Zhu D., Hu N., Schaefer D.W., Zarras P., Soucek M.D., Tiwari A. (2020). Water-based sol–gel coatings for military coating applications. Handbook of Waterborne Coatings.

[99] Li Z.F., Zhang C.J., Cui L., Zhu P., Yan C., Liu Y. (2017). Fire Retardant and Thermal Degradation Properties of Cotton Fabrics Based on APTES and Sodium Phytate through Layer-by-Layer Assembly. J. Anal. Appl. Pyrolysis.

[100] Graf E. (1983). Applications of Phytic Acid. J. Am. Oil Chem. Soc..

[101] Li S., Lin X., Li Z., Ren X. (2019). Hybrid Organic-Inorganic Hydrophobic and Intumescent Flame-Retardant Coating for Cotton Fabrics. Compos. Commun..

[102] Sudhakar Y.N., Selvakumar M., Bhat D.K., Sudhakar Y.N., Selvakumar M., Bhat D.K. (2018). Methods of Preparation of Biopolymer Electrolytes. Biopolymer Electrolytes.

[103] Pelton R. (2014). Polyvinylamine: A Tool for Engineering Interfaces. Langmuir.

[104] Magovac E., Vončina B., Budimir A., Jordanov I., Grunlan J.C., Bischof S. (2021). Environmentally Benign Phytic Acid-Based Nanocoating for Multifunctional Flame-Retardant/Antibacterial Cotton. Fibers.

[105] Liu X., Zhang Q., Peng B., Ren Y., Cheng B., Ding C., Su X., He J., Lin S. (2020). Flame Retardant Cellulosic Fabrics via Layer-by-Layer Self-Assembly Double Coating with Egg White Protein and Phytic Acid. J. Clean. Prod..

[106] Zeng T., Pignatello J.J., Li R.J., Mitch W.A. (2014). Synthesis and Application of a Quaternary Phosphonium Polymer Coagulant to Avoid N-Nitrosamine Formation. Environ. Sci. Technol..

[107] Carosio F., Alongi J., Malucelli G. (2013). Flammability and Combustion Properties of Ammonium Polyphosphate-/Poly(Acrylic Acid)-Based Layer by Layer Architectures Deposited on Cotton, Polyester and Their Blends. Polym. Degrad. Stab..

[108] Carosio F., Alongi J., Paravidino C., Frache A. (2017). Improving the Flame Retardant Efficiency of Layer by Layer Coatings Containing Deoxyribonucleic Acid by Post-Diffusion of Hydrotalcite Nanoparticles. Materials.

[109] Pan Y., Wang W., Liu L., Ge H., Song L., Hu Y. (2017). Influences of Metal Ions Crosslinked Alginate Based Coatings on Thermal Stability and Fire Resistance of Cotton Fabrics. Carbohydr. Polym..

[110] Donati I., Paoletti S., Rehm B.H.A. (2009). Material Properties of Alginates. Alginates: Biology and Applications.

[111] Pan Y., Liu L., Zhang Y., Song L., Hu Y., Jiang S., Zhao H. (2019). Effect of Genipin Crosslinked Layer-by-Layer Self-Assembled Coating on the Thermal Stability, Flammability and Wash Durability of Cotton Fabric. Carbohydr. Polym..

[112] Wang W., Guo J., Liu X., Li H., Sun J., Gu X., Wang J., Zhang S., Li W. (2020). Constructing Eco-Friendly Flame Retardant Coating on Cotton Fabrics by Layer-by-Layer Self-Assembly. Cellulose.

[113] Lazar S., Eberle B., Bellevergue E., Grunlan J. (2020). Amine Salt Thickening of Intumescent Multilayer Flame Retardant Treatment. Ind. Eng. Chem. Res..

[114] Van Langenhove L., Chapman R.A. (2013). Smart textiles for protection: An overview. Smart Textiles for Protection.

[115] An W., Ma J., Xu Q., Fan Q. (2020). Flame Retardant, Antistatic Cotton Fabrics Crafted by Layer-by-Layer Assembly. Cellulose.

[116] Zeng F., Qin Z., Li T., Chen Y., Yang L. (2020). Boosting Phosphorus–Nitrogen–Silicon Synergism through Introducing Graphene Nanobrick Wall Structure for Fabricating Multifunctional Cotton Fabric by Spray Assisted Layer-by-Layer Assembly. Cellulose.

[117] Li S., Lin X., Liu Y., Li R., Ren X., Huang T.S. (2019). Phosphorus-Nitrogen-Silicon-Based Assembly Multilayer Coating for the Preparation of Flame Retardant and Antimicrobial Cotton Fabric. Cellulose.

[118] Li P., Wang B., Liu Y.Y., Xu Y.J., Jiang Z.M., Dong C.H., Zhang L., Liu Y., Zhu P. (2020). Fully Bio-Based Coating from Chitosan and Phytate for Fire-Safety and Antibacterial Cotton Fabrics. Carbohydr. Polym..

[119] Lin D., Zeng X., Li H., Lai X. (2018). Facile Fabrication of Superhydrophobic and Flame-Retardant Coatings on Cotton Fabrics via Layer-by-Layer Assembly. Cellulose.

[120] Liu D., Zhang M., He L., Chen Y., Lei W. (2017). Layer-by-Layer Assembly Fabrication of Porous Boron Nitride Coated Multifunctional Materials for Water Cleaning. Adv. Mater. Interfaces.

[121] Safi K., Kant K., Bramhecha I., Mathur P., Sheikh J. (2020). Multifunctional Modification of Cotton Using Layer-by-Layer Finishing with Chitosan, Sodium Lignin Sulphonate and Boric Acid. Int. J. Biol. Macromol..

[122] Xue C.H., Wu Y., Guo X.J., Liu B.Y., Wang H.D., Jia S.T. (2020). Superhydrophobic, Flame-Retardant and Conductive Cotton Fabrics via Layer-by-Layer Assembly of Carbon Nanotubes for Flexible Sensing Electronics. Cellulose.

[123] Carosio F., Alongi J., Malucelli G. (2011). α-Zirconium Phosphate-Based Nanoarchitectures on Polyester Fabrics through Layer-by-Layer Assembly. J. Mater. Chem..

[124] Carosio F., Di Blasio A., Cuttica F., Alongi J., Frache A., Malucelli G. (2013). Flame Retardancy of Polyester Fabrics Treated by Spray-Assisted Layer-by-Layer Silica Architectures. Ind. Eng. Chem. Res..

[125] Apaydin K., Laachachi A., Ball V., Jimenez M., Bourbigot S., Ruch D. (2015). Layer-by-Layer Deposition of a TiO2-Filled Intumescent Coating and Its Effect on the Flame Retardancy of Polyamide and Polyester Fabrics. Colloids Surfaces Physicochem. Eng. Asp..

[126] Jordanov I., Magovac E., Fahami A., Lazar S., Kolibaba T., Smith R.J., Bischof S., Grunlan J.C. (2019). Flame Retardant Polyester Fabric from Nitrogen-Rich Low Molecular Weight Additives within Intumescent Nanocoating. Polym. Degrad. Stab..

[127] Wattanatanom W., Churuchinda S., Potiyaraj P. (2017). Intumescent Flame Retardant Finishing of Polyester Fabrics via the Layer-by-Layer Assembly Technique. Int. J. Cloth. Sci. Technol..

[128] Wattanatanom W., Charuchinda S., Potiyaraj P. (2019). Flame Behavior and Mechanical Properties of Polyester Fabrics Coated with Intumescent Coatings via Layer-by-Layer Assembly. Text. Res. J..

[129] Carosio F., Di Pierro A., Alongi J., Fina A., Saracco G. (2018). Controlling the Melt Dripping of Polyester Fabrics by Tuning the Ionic Strength of Polyhedral Oligomeric Silsesquioxane and Sodium Montmorillonite Coatings Assembled through Layer by Layer. J. Colloid Interface Sci..

[130] Pan Y., Liu L., Song L., Hu Y., Wang W., Zhao H. (2019). Durable Flame Retardant Treatment of Polyethylene Terephthalate (PET) Fabric with Cross-Linked Layer-by-Layer Assembled Coating. Polym. Degrad. Stab..

[131] Jiang Z., Wang C., Fang S., Ji P., Wang H., Ji C. (2018). Durable Flame-Retardant and Antidroplet Finishing of Polyester Fabrics with Flexible Polysiloxane and Phytic Acid through Layer-by-Layer Assembly and Sol-Gel Process. J. Appl. Polym. Sci..

[132] Yoshida E. (2010). Self-Assembly of Poly(Allylamine Hydrochloride) through Electrostatic Interaction with Sodium Dodecyl Sulfate. Colloid Polym. Sci..

[133] Apaydin K., Laachachi A., Ball V., Jimenez M., Bourbigot S., Toniazzo V., Ruch D. (2013). Polyallylamine-Montmorillonite as Super Flame Retardant Coating Assemblies by Layer-by Layer Deposition on Polyamide. Polym. Degrad. Stab..

[134] Apaydin K., Laachachi A., Ball V., Jimenez M., Bourbigot S., Toniazzo V., Ruch D. (2014). Intumescent Coating of (Polyallylamine-Polyphosphates) Deposited on Polyamide Fabrics via Layer-by-Layer Technique. Polym. Degrad. Stab..

[135] Kumar Kundu C., Wang W., Zhou S., Wang X., Sheng H., Pan Y., Song L., Hu Y. (2017). A Green Approach to Constructing Multilayered Nanocoating for Flame Retardant Treatment of Polyamide 66 Fabric from Chitosan and Sodium Alginate. Carbohydr. Polym..

[136] Kundu C.K., Wang X., Song L., Hu Y. (2018). Borate Cross-Linked Layer-by-Layer Assembly of Green Polyelectrolytes on Polyamide 66 Fabrics for Flame-Retardant Treatment. Prog. Org. Coat..

[137] Ziaur Rahman M., Kundu C.K., Nabipour H., Wang X., Song L., Hu Y. (2020). Hybrid Coatings for Durable Flame Retardant and Hydrophilic Treatment of Polyamide 6.6 Fabrics. Prog. Org. Coat..

[138] Jordanov I., Kolibaba T.J., Lazar S., Magovac E., Bischof S., Grunlan J.C. (2020). Flame Suppression of Polyamide through Combined Enzymatic Modification and Addition of Urea to Multilayer Nanocoating. J. Mater. Sci..

[139] Kundu C.K., Wang X., Song L., Hu Y. (2020). Chitosan-Based Flame Retardant Coatings for Polyamide 66 Textiles: One-Pot Deposition versus Layer-by-Layer Assembly. Int. J. Biol. Macromol..

[140] Mandal K., Ghose S., Mandal M., Majumder D., Talukdar S., Chakraborty I., Panda S.K., Das S., Dhara S. (2021). Notes on useful materials and synthesis through various chemical solution techniques. Chemical Solution Synthesis for Materials Design and Thin Film Device Applications.

[141] Alongi J., Carosio F., Malucelli G. (2012). Layer by Layer Complex Architectures Based on Ammonium Polyphosphate, Chitosan and Silica on Polyester-Cotton Blends: Flammability and Combustion Behaviour. Cellulose.

[142] Carosio F., Alongi J., Malucelli G. (2012). Layer by Layer Ammonium Polyphosphate-Based Coatings for Flame Retardancy of Polyester–Cotton Blends. Carbohydr. Polym..

[143] Haile M., Leistner M., Sarwar O., Toler C.M., Henderson R., Grunlan J.C. (2016). A Wash-Durable Polyelectrolyte Complex That Extinguishes Flames on Polyester-Cotton Fabric. RSC Adv..

[144] Leistner M., Abu-Odeh A.A., Rohmer S.C., Grunlan J.C. (2015). Water-Based Chitosan/Melamine Polyphosphate Multilayer Nanocoating That Extinguishes Fire on Polyester-Cotton Fabric. Carbohydr. Polym..

[145] Liu X., Meng X., Sun J., Tang W., Chen S., Peng X., Gu X., Fei B., Bourbigot S., Zhang S. (2020). Improving the Flame Retardant Properties of Polyester-Cotton Blend Fabrics by Introducing an Intumescent Coating via Layer by Layer Assembly. J. Appl. Polym. Sci..

[146] Liu L., Pan Y., Wang Z., Hou Y., Gui Z., Hu Y. (2017). Layer-by-Layer Assembly of Hypophosphorous Acid-Modified Chitosan Based Coating for Flame-Retardant Polyester-Cotton Blends. Ind. Eng. Chem. Res..

[147] Pan Y., Liu L., Wang X., Song L., Hu Y. (2018). Hypophosphorous Acid Cross-Linked Layer-by-Layer Assembly of Green Polyelectrolytes on Polyester-Cotton Blend Fabrics for Durable Flame-Retardant Treatment. Carbohydr. Polym..

[148] Wang B., Xu Y.J., Li P., Zhang F.Q., Liu Y., Zhu P. (2020). Flame-Retardant Polyester/Cotton Blend with Phosphorus/Nitrogen/Silicon-Containing Nano-Coating by Layer-by-Layer Assembly. Appl. Surf. Sci..

[149] Narkhede M., Thota S., Mosurkal R., Muller W.S., Kumar J. (2014). Layer-by-Layer Assembly of Halogen-Free Polymeric Materials on Nylon/Cotton Blend for Flame Retardant Applications. Fire Mater..

